# Downregulation of M Current Is Coupled to Membrane Excitability in Sympathetic Neurons Before the Onset of Hypertension

**DOI:** 10.1161/HYPERTENSIONAHA.120.15922

**Published:** 2020-10-12

**Authors:** Harvey Davis, Neil Herring, David J. Paterson

**Affiliations:** 1 From the Burdon Sanderson Cardiac Science Centre (H.D., N.H., D.J.P.), University of Oxford, United Kingdom; 2 Department of Physiology, Anatomy and Genetics, Wellcome Trust OXION Initiative in Ion Channels and Disease (H.D., D.J.P.), University of Oxford, United Kingdom; 3 Oxford Heart Centre, Oxford University Hospitals NHS Foundation Trust, John Radcliffe Hospital, United Kingdom (N.H.).

**Keywords:** hypertension, ion channels, primary dysautonomia, sodium, stellate ganglion

## Abstract

Neurohumoral activation is an early hallmark of cardiovascular disease and contributes to the etiology of the pathophysiology. Stellectomy has reemerged as a positive therapeutic intervention to modify the progression of dysautonomia, although the biophysical properties underpinning abnormal activity of this ganglia are not fully understood in the initial stages of the disease. We investigated whether stellate ganglia neurons from prehypertensive SHRs (spontaneously hypertensive rats) are hyperactive and describe their electrophysiological phenotype guided by single-cell RNA sequencing, molecular biology, and perforated patch clamp to uncover the mechanism of abnormal excitability. We demonstrate the contribution of a plethora of ion channels, in particular inhibition of M current to stellate ganglia neuronal firing, and confirm the conservation of expression of key ion channel transcripts in human stellate ganglia. We show that hyperexcitability was curbed by M-current activators, nonselective sodium current blockers, or inhibition of Na_v_1.1-1.3, Na_v_1.6, or I_NaP_. We conclude that reduced activity of M current contributes significantly to abnormal firing of stellate neurons, which, in part, contributes to the hyperexcitability from rats that have a predisposition to hypertension. Targeting these channels could provide a therapeutic opportunity to minimize the consequences of excessive sympathetic activation.

## Abstract 2

Supplemental Digital Content is available in the text.

Sympathetic hyperactivity is known to contribute to the pathophysiology of a variety of cardiovascular diseases, including hypertension, myocardial infarction, and heart failure, although the cellular mechanisms responsible for this have not been fully elucidated.^1,2^ In hypertension, increased cardiac sympathetic drive is linked to the development of left ventricular hypertrophy^3^ and subsequent heart failure, which are independent predictors of mortality.^4^

A significant component of this sympathetic hyperactivity resides at the level of the postganglionic sympathetic neuron, in particular, the stellate ganglia, which contain the cell bodies that predominantly innervate the heart.^5^ These cells have enhanced Ca^2+^ driven^6,7^ norepinephrine^8^ and epinephrine release^9^ and impaired reuptake via the Norepinephrine transporter.^10^ However, both animal models and patients with hypertension also have increased sympathetic nerve firing rate as measured by muscle and renal sympathetic nerve activity.^11^ Whether this is centrally driven or results from changes in the excitability of postganglionic neurons before the onset of hypertension is unknown,^12^ as are the specific ion channels involved in the sympathetic phenotype. Early work has reported enhanced electrical excitability in another sympathetic ganglia (superior cervical ganglia [SCG]) in the SHR (spontaneously hypertensive rat), although conflicting reports suggest the neural phenotype might be related to changes in either K_Ca_ or A-type potassium current.^13,14^

We used single-cell RNA sequencing (scRNAseq) analysis of stellate ganglia neurons to guide a detailed biophysical characterization in the prehypertensive SHR,^15^ to test the hypothesis that several *a priori* ion channels, which are conserved in human stellate ganglia tissue, underpin the neural phenotype in the prediseased state. Here, we highlight the role of a reduction in M current (I_M_)—a noninactivating inhibitory K^+^ current, which provides a restriction upon firing^16^—as a putative target for pharmacological intervention to reduce sympathetic activity.

## Methods

An expanded Materials and Methods section is available in the Data Supplement for patch clamp, scRNAseq, real time quantitative polymerase chain reaction, and immunohistochemistry. The single-cell sequencing dataset generated during this study is available at genome expression omnibus (GSE144027). Further data and details on materials and protocols related to this study are also available upon reasonable request from the corresponding authors.

### Clinical Samples

Stellate ganglia were collected from organ donors at the time of organ procurement as approved by the University of California Los Angeles institutional review board: 12-000701. Written informed consent was provided by the patient or appropriate designee. This study complies with the Declaration of Helsinki.

### Animals

Animal use complied with the University of Oxford Local Ethical Guidelines and was in accordance with the Guide for the Care and Use of Laboratory Animals published by the US National Institutes of Health (Publication No. 85-23, revised 2011) and the Animals (Scientific Procedures) Act 1986 (United Kingdom). Experiments were performed under British Home Office Project License (PPL 30/3131 [D.J.P.] and P707EB251 [D.J.P.]). All animals were ordered from Envigo and housed on a 12-hour day-night cycle. Animals were euthanized via an overdose of pentobarbitone and confirmed via exsanguination according to Schedule 1 of the Animals (Scientific Procedures) Act 1986 (United Kingdom). Male normotensive Wistar rats and prehypertensive SHRs were culled at 5 to 6 weeks of age, at which age SHRs possess a phenotype unaffected by prolonged hypertension as found in older animals. Moreover, around this age, postnatal ion channel expression stabilizes in sympathetic ganglia.^17^

### Statistical Analysis

All datasets were normality tested, except for firing rate, which was taken as a discontinuous variable and treated as nonparametric data. Statistical analysis and normality tests were performed in Graphpad Prism (v8.2.1). The specific statistical test applied is stated in the figure legends with statistical significance accepted at *P*<0.05 on 2-tailed tests.

## Results

### Stellate Ganglia Neurons Are Hyperexcitable in the Prehypertensive SHR

**Figure 1.**
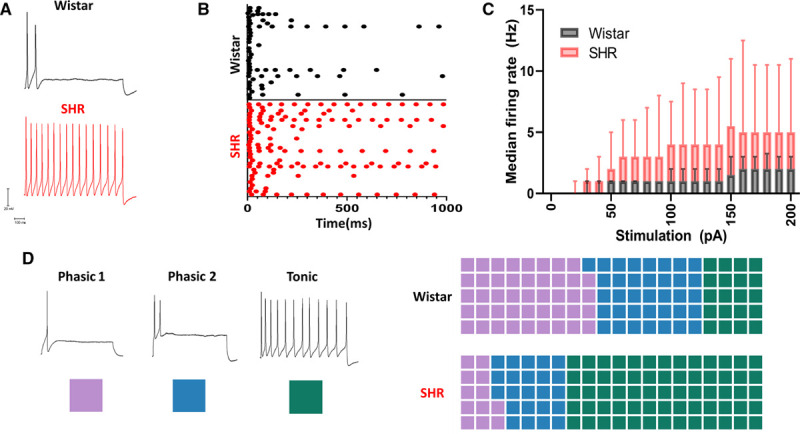
Stellate ganglia neurons of the spontaneously hypertensive rat have a hyperactive phenotype with an altered electrophysiological profile as observed by perforated patch-clamp recordings. **A**, Example traces showing the response of sympathetic neurons from control Wistar and prehypertensive SHRs (spontaneously hypertensive rats) to 150 pA of current injection for a duration of 1000 ms. **B**, Time course of induced firing following a 1000-ms 150-pA current injection in 30 example Wistar and SHR neurons. **C**, Median response of Wistar (black) and SHR (red) neurons to a range of current injections (Wistar, n=66; SHR, n=69; mixed-effects model: *P*<0.0001). **D**, Examples of sympathetic neuron firing rates are identified by their classical nomenclature. Here, the color scheme used to indicate these subtypes throughout the article is indicated by a colored square. A trend toward tonic firing neurons was observed in the SHR, shown as percentage of cells conforming to each subtype (Wistar, n=66; phasic 1, n=29; phasic 2, n=24; tonic, n=13; SHR, n=73; phasic 1, n=9; phasic 2, n=17; tonic, n=47; χ^2^=12.37, df=2, *P*=0.0021).

Perforated patch-clamp measurements demonstrate that stellate ganglia neurons from prehypertensive SHRs have a significantly higher firing rate than neurons from normotensive age-matched Wistars as shown in Figure 1A and 1C. This firing rate difference appears to be time resolved, with the majority of Wistar neurons firing action potentials within only the first 300 ms of stimulation. This phenotype is represented in Figure 1B via a raster plot of 30 Wistar and SHR neurons during a 1000-ms 150-pA current injection. We also observed other indicators of cellular hyperexcitability. The change in firing rate was accompanied by a 3.1±1.1-mV depolarization of resting membrane potential between Wistar and SHR neurons (unpaired *t* test, *P*=0.0072). The rheobase (minimum current injection of duration >300 ms required to reach the action potential threshold) was also decreased in SHR neurons (−20 pA; Mann-Whitney *U* test; *P*=0.0013) when measured by a series of 10 pA current steps of duration 1000 ms in the range 0 to 200 pA in amplitude.

### SHR Has a Higher Percentage of Tonic Firing Neurons

Neuronal firing behavior has been previously characterized into 3 firing property classifications.^18^ Phasic 1 firing neurons represent neurons that fire only one action potential during a 1000-ms current injection in the range of 0 to 200 pA. Phasic 2 firing neurons fire 2 to 5 action potentials, all within the first 500 ms of a 1000-ms stimulation pulse for all recordings within the stimulation range 0 to 200 pA. Tonic firing neurons fire either >6 action potentials or continue to fire after 500 ms of stimulation of a 1000-ms stimulation pulse within the current injection range 0 to 200 pA. In Figure 1D and subsequent figures, these classes have been assigned color codes to allow for a visual representation of the firing rate and time course of firing. When the percentage of neurons conforming to these classes is compared between Wistar and SHR neurons (Figure 1E), Wistar neurons were found to be predominantly phasic 1 and phasic 2 firing, whereas in the SHR, neurons were predominantly found to exhibit tonic firing.

### Change in Ion Channel Subunit Expression Was Observed in SHR Neurons

**Figure 2.**
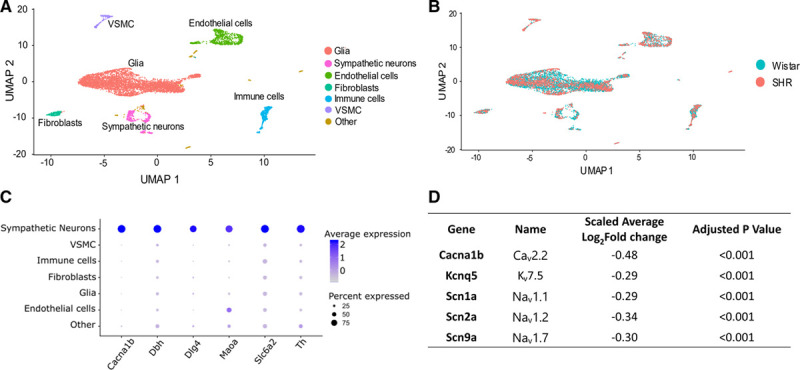
Single-cell RNA sequencing on Wistar and SHR (spontaneously hypertensive rat) populations reveals altered channel subunit expression in SHR stellate ganglia neurons. **A**, Single-cell RNA sequencing reveals multiple clusters of cell transcriptomes. **B**, These cell clusters align to multiple cell types, as defined using the best-known markers for these cell types. **C**, Markers used for sympathetic neurons are shown to be primarily expressed in sympathetic neurons (*Cacna1b*, Ca_v_2.2; *Dbh*, dopamine-β-hydroxylase; *Dlg4*, PSD-95; *Maoa*, monoamine oxidase A; Slc6a2, Norepinephrine transporter; *Th* [tyrosine hydroxylase]). **D**, Differential expression analysis of key ion channels in Wistar vs SHR neurons. Differential expression analysis performed using MAST in the Seurat package reveals a number of ion channel subunits are downregulated at transcriptional level in the SHR. UMAP indicates uniform manifold approximation and projection; and VSMC, vascular smooth muscle cell.

To investigate the underlying ion channel differences responsible for the difference in firing rate between SHR and Wistar stellate neurons, we undertook an scRNAseq analysis of the ganglia. scRNAseq revealed a heterogeneous population of cell clusters in the both Wistar and SHR dissociated stellate ganglia (Figure 2A), which mapped to known cell type markers (Figure 2B) including 2 distinct population of cells that are specific for the full range of known sympathetic markers (Figure 2C; Figure S5C in the Data Supplement). The main difference between these 2 sympathetic neuronal populations is that one also expressed the cotransmitter neuropeptide Y. Differential expression analysis between the Wistar and SHR sympathetic neuron populations identified in Figure 2C highlight a significant decrease in 5 ion channel subunit encoding genes that may contribute to firing rate of stellate ganglia sympathetic neurons Figure 2D (full list of tested genes in Figure S6D).

### M Current Is Functionally Reduced in SHR Stellate Ganglia Neurons and Transcript Present in Human Stellate Ganglia

**Figure 3.**
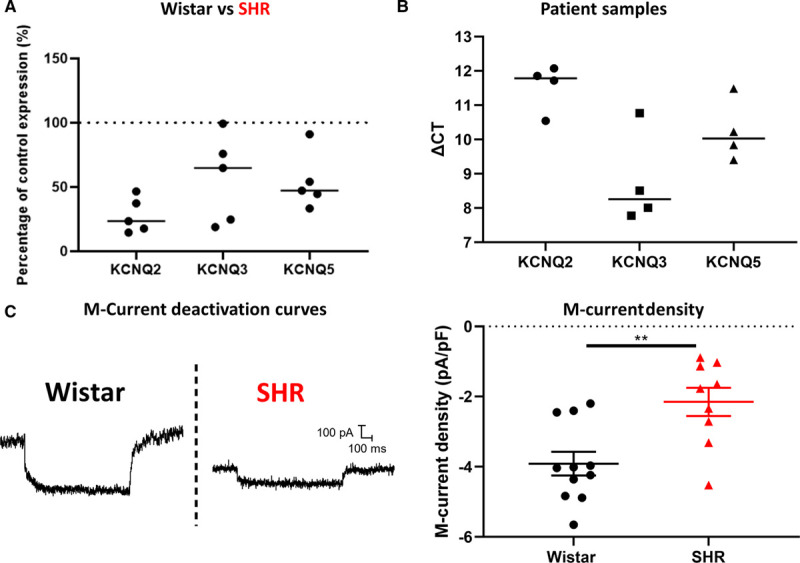
M current is downregulated at a functional level in the stellate ganglia, and pharmacological manipulation of M-current can reverse or induce the firing rate phenotype observed in the SHR (spontaneously hypertensive rat). **A**, real time quantitative polymerase chain reaction reveals a downregulation of KCNQ2 (K_v_7.2), KCNQ3 (K_v_7.3) and KCNQ5 (K_v_7.5) expression in the left stellate ganglia of 3- to 6-wk-old prehypertensive SHR (median; percentage decrease: KCNQ2, 76.58%; KCNQ3, 35.16%; KCNQ5, 52.87%; Wistar, n=8; SHR, n=5). Individual SHR data points are shown as a percentage of the average of Wistar controls. **B**, KCNQ2, KCNQ3, and KCNQ5 subunit expression was confirmed in patient samples of stellate ganglia, taken from donors (median; ΔCt: KCNQ2, 11.78; KCNQ3, 8.259; KCNQ5, 10.03; n=4). **C**, M-current density was revealed to be decreased in the SHR as determined by a decrease in XE-991 sensitive current, via a step protocol from −25 to −55 mV (median±IQR; Wistar, −4.032 pA/pF, n=11; SHR, −1.770 pA/pF, n=9; Mann-Whitney *U* test: *P*=0.0074). Raw data traces of representative deactivation protocols from Wistar and SHR neurons are shown.

Was a decrease in I_M_ subunit expression the most likely explanation for the difference in phenotype? When assessed by real time quantitative polymerase chain reaction, gene expression of I_M_ encoding KCNQ2, KCNQ3, and KCNQ5 subunits was decreased in total RNA extracted from whole SHR ganglia (Figure 3A). We also confirmed I_M_ subunit expression in samples of total RNA taken from human stellate ganglia (Figure 3B). Using immunohistochemistry, I_M_ encoding subunits KCNQ2, KCNQ3, and KCNQ5 were also shown to be expressed at a protein level in TH (tyrosine hydroxylase)-positive cells (Figure S3)—a classic marker for sympathetic neurons.

I_M_ was analyzed through deactivation curves, in this case applied from a holding potential of −25 to −55 mV, allowing for a relaxation of current corresponding to I_M_. These recordings were made in perforated patch, as I_M_ is known to rundown in whole-cell recordings. To confirm that the current measured was I_M_, we measured current that was inhibited by the I_M_ inhibitor 10 µmol/L XE-991.^19^ These data were then normalized to cell capacitance. By this measure, I_M_ was shown to be functionally present in stellate ganglia neurons and to be downregulated in SHR relative to Wistar (Figure 3C).

### I_M_ Inhibition Increased the Excitability of Wistar Stellate Ganglia Neurons

**Figure 4.**
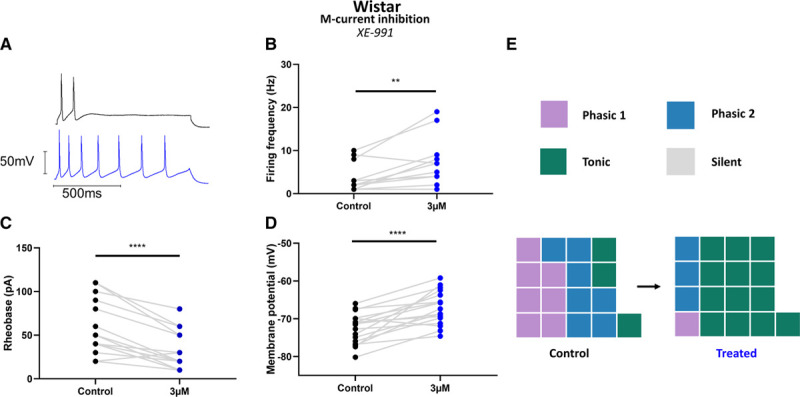
M-current inhibition can recapitulate the SHR (spontaneously hypertensive rat) phenotype in Wistar neurons. **A**, Example trace of M-current inhibition by 3 µmol/L XE-991 in a Wistar neuron, shown before (black) and after (blue) treatment. **B**, M-current inhibition by 3 µmol/L XE-991 significantly increased maximum firing rate induced by current injections in the range 10 to 200 pA (median; control, 3 Hz; treated, 7 Hz; Wilcoxon test: n=12, *P*=0.0059). **C**, M-current inhibition by 3 µmol/L XE-991 caused a depolarization of Wistar neuron resting membrane potential (mean±SEM; control, −72.59±0.97 mV; 3 µmol/L XE-991, −67.07±1.08 mV; paired *t* test: n=17, *P*<0.0001). **D**, M-current inhibition by 3 µmol/L XE-991 decreased Wistar neuron rheobase (median; control, 50 pA; 3 µmol/L XE-991, 20 pA; Wilcoxon test: n=17, *P*<0.0001). **E** and **J**, A graphical representation of subtype changes following the application of M-current inhibitors; (**E**) 3 µmol/L XE-991 in Wistar neurons. A trend toward a predominantly tonic firing subtype was observed in both cases.

I_M_ pharmacology was used to assess its role in firing rate alongside other electrophysiological parameters in stellate ganglia neurons. I_M_ inhibition by 3 µmol/L XE-991^19^ caused a significant increase in Wistar stellate ganglia neuron firing rate, measured as the maximum firing rate of tested neurons within a stimulation range of 0 to 200 pA (Figure 4B). This was accompanied by depolarization of the resting membrane potential (Figure 4C) and a significantly decreased rheobase (Figure 4D). Comparable results were seen after application of 30 µmol/L linopirdine—an alternative I_M_ inhibitor at a dose comparable in efficacy to 3 µmol/L XE-991^20^ (data not shown).

### I_M_ Activation Reduced Excitability of SHR and Wistar Stellate Ganglia Neurons

**Figure 5.**
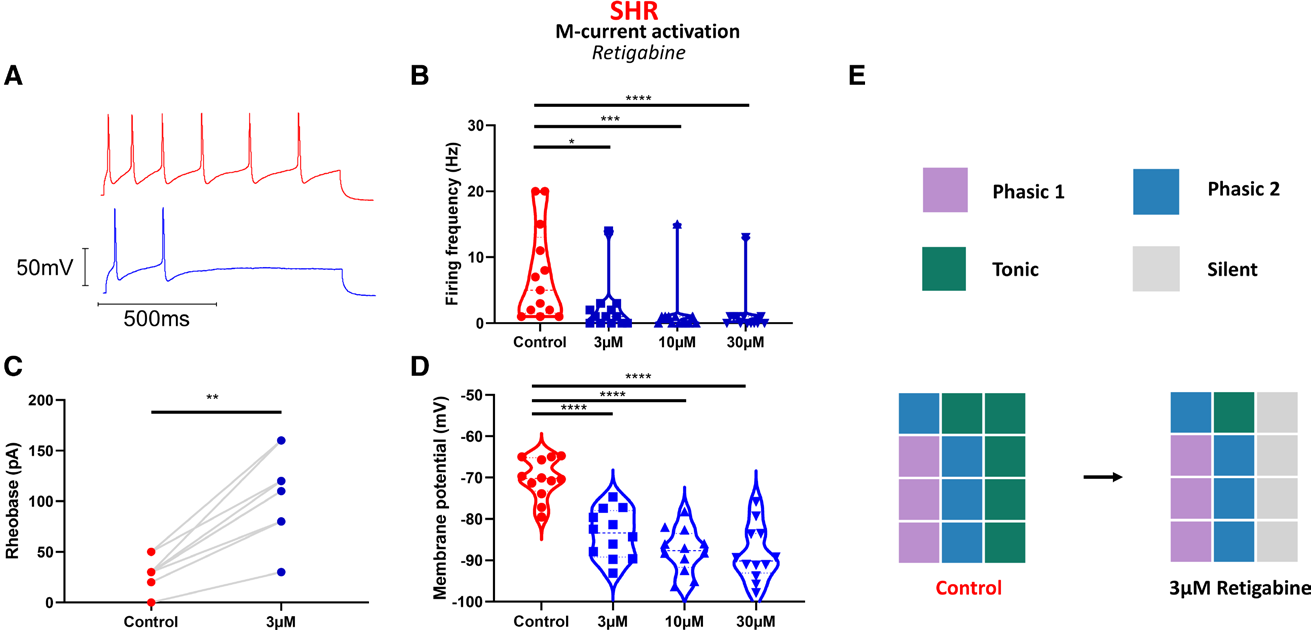
M-current activation in SHR (spontaneously hypertensive rat) neurons reduces aberrant electrical activity. **A**, M-current activation in SHR neurons by retigabine significantly reduced maximum firing rate at all tested doses (median; control, 5 Hz; 3 µmol/L retigabine, 1 Hz; 10 µmol/L retigabine, 0 Hz; 30 µmol/L retigabine, 0 Hz; Friedman test: n=13, *P*<0.0001; Dunn multiple comparisons: control vs 3 µmol/L, *P*=0.018; control vs 10 µmol/L, *P*=0.0002; control vs 30 µmol/L, *P*<0.0001). **C**, M-current activation by 3 µmol/L retigabine increased SHR rheobase (median; control, 30 pA; 3 µmol/L retigabine, 110 pA; Wilcoxon test: n=9, *P*=0.0039). **D**, M-current activation by retigabine causes a hyperpolarization of resting membrane potential at higher doses (mean±SEM; control, −70.27±1.39 mV; 3 µmol/L, −83.63±1.67 mV; 10 µmol/L, −87.69±1.53 mV; 30 µmol/L, −88.45±1.91 mV; 1-way ANOVA: n=12, *P*<0.0001; Dunnett multiple comparisons: control vs 3 µmol/L, *P*<0.0001; control vs 10 µmol/L, *P*<0.0001; control vs 30 µmol/L, *P*<0.0001). **E**, A graphical representation of the effect of M-current activator retigabine on SHR firing rate, which prevents firing in a third of tested neurons (gray squares).

Since I_M_ was shown to be reduced, but not entirely absent via 10 µmol/L XE-991–sensitive deactivation curves (Figure 3C), we also tested whether increasing SHR I_M_ via the activator retigabine^21^ would be sufficient to reduce firing rate. We found that retigabine significantly reduced SHR stellate ganglia neuron maximum firing rate at all 3 doses tested (Figure 5B). These data were visualized as firing rate subtypes in Figure 5E, where the number of tonic neurons decreases, and 4 cells were prevented from firing at 3 µmol/L retigabine in the stimulation range 0 to 200 pA. Retigabine increased rheobase amplitude at 3 µmol/L in neurons that still fired within 0 to 200 pA stimulation (Figure 5C), but at higher doses too few neurons still fired in this range to allow for a quantitative comparison of rheobase amplitude. These observations were accompanied by a hyperpolarization of the resting membrane potential at all tested doses (Figure 5D).

### I_Na_, SK Channels, and K_v_2.1 Also Modulate SHR Stellate Neuron Firing Rate

Using scRNAseq and a series of pharmacological inhibitors, we screened a range of ion channels with a known role in determining the firing rate of other neuronal populations. In Figure S2, the patterns of expression for key channels implicated in firing rate are shown against cell populations highlighted in Wistar and SHR stellate ganglia. These channel subunits were chosen based upon the expression of these subunits in stellate ganglia neurons, availability of selective pharmacology, and a demonstrated role in firing rate in other neuronal populations. For the chosen channels, either 1 or 2 pharmacological inhibitors were applied to SHR neurons to observe any effect on firing rate (Figures 4 through 6; Figure S2). Pharmacological inhibitors that had a significant effect on the firing rate of these neurons are shown in Figures 4 and 5, and nonsignificant inhibitors are shown in Figure S2.

Low-dose (10 nmol/L) tetrodotoxin^22^ significantly reduced firing rate in SHR neurons (Figure 4D). Two compounds tested, K_v_2.1-2.2 inhibitor, guangxitoxin,^23^ and SK channel inhibitor, apamin,^24^ increased SHR stellate ganglia neuron firing rate (Figure S2).

### Specific Na_v_ Subunit Inhibitors Reduce SHR Stellate Neuron Firing Rate

**Figure 6.**
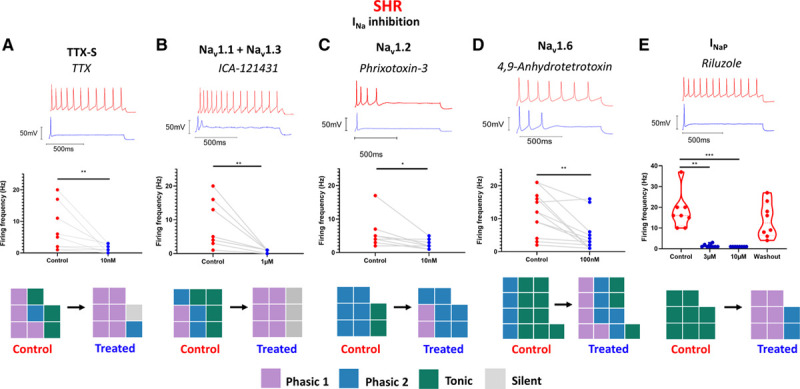
An exploration of the role of Na_V_ subtypes in SHR (spontaneously hypertensive rat) firing rate. The effect of specific Na_V_ subtype and conductance state inhibitors were investigated in SHR stellate ganglia neurons. **A**, Nonselective tetrodotoxin (TTX)-sensitive Na_v_ inhibition by low-dose (10 nmol/L) TTX significantly reduced SHR neuron maximum firing (median; control, 2 Hz; treated, 1 Hz; Wilcoxon test: n=14, *P*=0.0078). **B**, Na_v_1.1 inhibition by 1 µmol/L ICA-121431 in SHR neurons significantly reduced maximum firing rate in SHR neurons (median; control, 4 Hz; treated, 1 Hz; Wilcoxon test: n=9, *P*=0.0039). **C**, Na_v_1.2 inhibition by 10 nmol/L phrixotoxin-3 in SHR neurons significantly reduced maximum firing rate in SHR neurons (median; control, 4.5 Hz; treated, 2 Hz; Wilcoxon test: n=8, *P*=0.0313). **D**, Nav1.6 inhibition by 100 nmol/L 4,9-anhydrotetrodotoxin significantly reduced maximum firing rate in SHR neurons (median; control, 12 Hz; treated, 3 Hz; Wilcoxon test: n=13, *P*=0.0015). **E**, I_NaP_ inhibition by 3- to 10-µmol/L riluzole in SHR neurons reduced firing rate (median; control, 16 Hz; 3 µmol/L, 1 Hz; 10 µmol/L, 1 Hz; washout, 12.5 Hz; Friedman test: n=8, *P*<0.0001; Dunn multiple comparisons test: control vs 3 µmol/L, *P*=0.003; control vs 10 µmol/L, *P*=0.007).

Of these identified targets, I_Na_ was of the most interest due to its strong control over firing rate. Using a panel of selective inhibitors, we further investigated the role of individual Na_V_ subunits that were demonstrated to be present in the stellate ganglia via scRNAseq (Figure S2). The Na_v_1.1 and Na_v_1.3 inhibitor ICA-121431^25^ was tested for its effect on SHR stellate ganglia neuron firing rate. One μmol/L ICA-121431 significantly reduced SHR firing rate and converted a majority of neurons to phasic 1 firing (Figure 6B). The Na_v_1.2-1.3 and Na_v_1.5 inhibitor phrixotoxin-3 is specific for Na_v_1.2 at the tested dose, 10 nmol/L, and was, therefore, utilized as a selective Na_v_1.2 inhibitor.^26^ Ten nmol/L phrixotoxin-3 significantly reduced the SHR neuron firing rate and prevented tonic firing (Figure 6C). Inhibition of Nav1.6 by 4,9-anhydrotetrodotoxin^27^ significantly reduced firing of SHR neurons (Figure 6D). We also tested 100 nmol/L 4,9-anhydrotetrotoxin^27^ on XE-991–inhibited Wistar neurons and found that, similar to in the SHR neurons, it significantly decreased firing rate and converted the majority of the tested neurons to phasic 1 (Figure S5). The persistent Na_v_ channel inhibitor riluzole^28^ inhibited firing in the tested population and reduced most neurons to phasic 1 at the lowest dose tested, 3 µmol/L (Figure 6E).

Membrane depolarization can limit Na_v_ availability through increasing Na_V_ inactivation.^29^ To ensure the depolarized resting membrane potential observed in the SHR did not limit SHR firing rate, we applied a series of negative 1-s current injections in the range −10 to −100 pA followed immediately by a stimulatory 1-s 150-pA current injection. By this method, we found that there was no significant difference between these current steps or a 0-pA control prepulse in SHR neurons (Figure S1G).

### Tonic Neurons Have Less I_M_ and More I_Na_

By studying action potential kinetics and I_M_ density between firing rate classes, we aimed to gain further insight into the involvement of I_M_ and I_Na_ in the origin of phasic 1, phasic 2, and tonic firing classes (Figure S4). First, to highlight any differences in I_Na_ between subtypes, we used whole-cell recordings of single action potentials to measure action potential upstroke and action potential amplitude. These data revealed higher I_Na_ availability in tonic and phasic 2 populations than in phasic 1 as measured by amplitude (Figure S3D) or upstroke (Figure S4C). When viewed per firing patterns, we observed significantly less I_M_, as determined by deactivation curves, in tonic firing neurons than phasic 2 neurons (Figure S4B).

## Discussion

We report four primary novel findings. First, sympathetic stellate ganglia neurons from the prehypertensive SHR are hyperexcitable, which manifests as a higher firing rate, depolarized resting membrane potential, and reduced rheobase. Second, I_M_ is downregulated in the stellate ganglia neurons of the SHR, and this is the causative mechanism for membrane hyperexcitability. Third, KCNQ2, KCNQ3, and KCNQ5 transcripts for I_M_ are conserved in human stellate ganglia providing a contextualized basis for their potential wider physiological role. Finally, hyperexcitability can be curbed either by elevation of remaining I_M_ or via reduction of I_Na_ (nonselective or selective inhibition of Na_v_1.1–1.3, Na_v_1.6, or I_NaP_).

Previous work in the SHR model has reported repetitive firing in neurons of the SCG.^13,14,30^ Support for the role for K_Ca_ currents was based upon increased firing rate following K_Ca_ reduction using the SK inhibitor apamin or nonselective K^+^ channel inhibitor Tetraethylammonium.^14^ Similarly, we report a role for SK calcium-activated channels in fine-tuning SHR stellate neuron firing rates, as shown by the increased firing rate after apamin inhibition of SK channels (Figure S2), although our data suggest that this is not a disease causative mechanism. We observed no change in these subunits via single-cell sequencing in stellate neurons (Figure 2D) and have previously reported that membrane Ca^2+^ currents from SCG and stellate ganglia neurons are increased in the SHR model,^6,7^ which would not favor reduced calcium-activated potassium currents. Therefore, when all data are taken together, evidence would not favor reduced calcium-activated potassium currents playing a significant role in sympathetic postganglionic hyperactivity in this model.

Increased excitability might also be due to changes in A-type K^+^ current, which was reported to be larger in SCG neurons of the SHR.^13^ However, it is worth noting that A-type K^+^ current is similar in phasic and tonic neurons of the SCG,^31^ and changes in A-type K^+^ current would not be expected to influence membrane potential, as it is by definition a high-voltage-activated current. We also observed no changes in A-type K^+^ current encoding transcript expression (Figure 2D; Figure S2D) between SHR and Wistar stellate neurons, suggesting this ion channel is unlikely to explain the phenotype described here.

### Role of I_M_

An early study by Yarowsky and Weinreich^30^ observed that muscarine increased firing rate in SHR SCG neurons, from which they suggest that I_M_ is not downregulated. This observation differs from our results in stellate ganglia neurons where we directly measured I_M_, and may reflect either ganglia specificity, or that a difference in I_M_ was nondetected by their methodology. The increased firing rate we observe in the SHR (Figure 1) fits well with the known characteristics of I_M_.^16^ Its role is consistent with both a general increase in firing rate (Figure 1A and 1B) and a loss of time-resolved firing patterns (Figure 1B). I_M_ has a powerful effect on neuronal resting membrane potential,^32^ as demonstrated in Figures 4 and 5. The loss of I_M_ in the SHR is probably directly related to the depolarization of the resting membrane potential (Figure 1D). Notably, reversing this depolarization does not alter firing rate itself (Figure S1G) and is only associated with I_M_ loss, rather than a cause of hyperactivity. Finally, I_M_ inhibition also reduces the rheobase (Figure 4D and 4I), which is consistent with the phenotype observed (Figure 1E), and with the idea for I_M_ downregulation being a causative factor of stellate neuronal hyperactivity in the SHR. Does this translate *in vivo*? Prior work has investigated the systemic effect of I_M_ modulators *in vivo* in Wistar and SHR rodents.^33,34^ I_M_ activation by retigabine appears to reduce increased plasma catecholamines in the SHR,^34^ consistent with I_M_ downregulation driving sympathetic pathology. Moreover, I_M_ inhibition by XE-991 had a greater effect on cardiac output in the Wistar than the SHR in line with reduced I_M_ in SHR sympathetic neurons.^34^ However, I_M_ is expressed in a range of tissues including the vasculature, brain, and sympathetic neurons but is notably absent from the heart.^35^ Our data highlight the role of I_M_ in dysautonomia associated with the prehypertensive state. The data are also consistent with prior genome wide association studies, which noted that single nucleotide polymorphisms in KCNQ5 (rs12195276-T) and KCNQ3 (rs138693040-T) are associated with pulse pressure^36^ and long QT syndrome,^37^ respectively. Therefore, further study is warranted to comprehensively isolate and assess the contribution of sympathetic I_M_ to the cardiac and systemic phenotypes of hypertension *in vivo*.

### Role of I_Na_

As observed for other neuron populations,^38^ I_Na_ modulation also appears to be a powerful regulator of firing rate in SHR stellate ganglia neurons (Figures 4D and 5). The pattern of expression for Na_v_ subunits is different than that of sensory or central neurons, with a stellate ganglia neuron expressing Na_v_1.1 to 1.3 and Na_v_1.6 to 1.7 (Figure 4A), which might have translational utility. Specifically, there are case reports of thoracic epidural anesthesia (using Na^+^ channel blockers) being successfully used in patients experiencing recurrent, life-threatening ventricular tachycardias to block sympathetically driven arrhythmia as a bridge to surgical cardiac sympathetic denervation via stellectomy.^1,39^

### I_Na_ and I_M_ in Electrophysiological Subpopulations

Others have suggested a relationship between reduced I_M_^31,40–42^ and higher I_Na_^41^ in determining SCG neuron firing types. We expand upon observations made by Luther and Birren,^41^ to include phasic 1 and phasic 2 firing neurons and in so doing demonstrate that I_Na_ is lower only in phasic 1 firing neurons. While downregulation of I_M_ provides an explanation for membrane hyperactivity, we have not identified a pathway by which this may originate. One possible explanation is that this results from continual presynaptic input, which contributes to ganglionic long term potentiation.^43^ It is also possible that other channels may be contributing to this phenotypic difference, but as the measured variables all correlate well with our observations of I_M_ inhibition in Wistar neurons, it seems likely that I_M_ downregulation alone is the major cause.

### Limitations

This study has several limitations. First, the SHR model of hypertension has a genetic basis, as well as being a rodent model, which differs in several respects from humans,^44^ although we have confirmed that I_M_ subunits are expressed in human stellate ganglia indicating conservation of transcript that provides a basis for a translational role for this ion channel (Figure 3B). Second, the stellate ganglia contain a heterogeneous population, with both cardiac and noncardiac innervating neurons. However, previous reports from the Wistar stellate ganglia report no differences in baseline membrane properties between neurons with different innervation targets.^42^ Third, RNA sequencing does not establish whether transcripts encode proteins that confer physiological function, although we provide a large quantity of electrophysiological data to reinforce observations made by this technique. scRNAseq was the best approach for this study, as bulk sequencing would also incorporate contaminating cell types, for example, vascular cells, which are known to have ion channel expression changes in the SHR.^45^ It should be noted that we observed downregulation of all 3 I_M_ encoding subunits via RT-qPCR but only downregulation of KCNQ5 by scRNAseq. For KCNQ2, the detected expression level was relatively low via scRNAseq (Figure 2C). Finally, culturing neurons may change neuronal phenotype. To reduce the impact of culturing upon the electrical phenotype of stellate ganglia neurons, we cultured for a relatively short period of 1 to 5 days in vitro, during which we observed no time-dependent effect upon firing rate (Figure S1A). Further to this, our data are comparable to prior intracellular recordings of Wistar stellate ganglia neurons in situ.^42^ Beyond a decrease in transcript expression, we have not addressed the causative mechanism for the I_M_ downregulation in this article. Speculatively, this may be downstream of enhanced presynaptic activity, resulting from the long-term potentiation of the postsynaptic sympathetic neuron. This mechanism has previously been described in SHR SCG,^46^ although it has not yet been directly linked to either changes in membrane properties or I_M_ expression.

### Perspectives

In conclusion, we have described a phenotype of sympathetic hyperactivity in stellate ganglia neurons of the SHR and provided an electrophysiological framework for this observation, guided by scRNAseq of the stellate ganglia, with human validation of key transcripts. Targeting certain ion channels in the stellate ganglia, such as I_M_, may provide a reversible therapeutic opportunity to treat cardiac sympathetic hyperresponsiveness over and above interventions like surgical stellectomy.

## Acknowledgments

We acknowledge the High-Throughput Genomics Group at the Wellcome Trust Centre for Human Genetics for producing the single-cell RNA sequencing data and for the initial cell-ranger analysis. We would like to acknowledge Olujimi A. Ajijola, MD, and his team for providing human stellate ganglia for this study. H. Davis performed, interpreted, and analyzed all experiments. H. Davis produced figures. H. Davis, N. Herring, and D.J. Paterson wrote and edited the manuscript. H. Davis, N. Herring, and D.J. Paterson designed the project.

## Sources of Funding

We acknowledge the British Heart Foundation (RG/17/14/33085), the British Heart Foundation Center of Research Excellence (CRE Oxford), the Wellcome Trust OXION Program (102161/Z/13/Z), and the Medical Sciences Doctoral Training Centre, University of Oxford (BST0008Z) for funding this work. N. Herring is supported by a British Heart Foundation Intermediate Fellowship (FS/15/8/3115).

## Disclosures

None.

## Supplementary Material



## Novelty and Significance

### What Is New?

- We have identified electrophysiological hyperactivity in prehypertensive SHRs (spontaneously hypertensive rats) stellate ganglia neurons, alongside highlighting the molecular and transcriptional phenotype for this change.
- We also highlight multiple mechanisms to curb this increased sympathetic hyperactivity, via either Na_v_- or I_M_-mediated pharmacology.
- We provide a comprehensive single-cell sequencing dataset for the SHR stellate ganglia, highlighting changes in neuron-specific gene expression during hypertension, and further show key transcripts are conserved in human stellate ganglia.

### What Is Relevant?

- We have identified M-current downregulation as a mechanism for sympathetic neuron hyperactivity in prehypertension, which is likely to contribute to sympathetic dysautonomia.
- Targeting either M current or the specific sodium channel subtypes may provide novel therapeutic avenues for the reduction of sympathetic hyperactivity in hypertension.

### Summary

We identify a hyperactive phenotype in stellate ganglia neurons of the prehypertensive SHR, which we demonstrate results from a transcriptional and functional downregulation of M current. To do so, we use a combination of perforated and whole-cell patch-clamp electrophysiology, single-cell RNA sequencing, real time quantitative polymerase chain reaction, and immunohistochemistry. This phenotype correlates with in vivo observations of increased nerve firing in hypertension. We also identify mechanisms for curbing this hyperactivity, via either inhibition of Na_v_ subunits identified by single-cell RNA sequencing, inhibition of persistent Na_v_ current, or activation of M current by retigabine. These targets may prove useful to reduce or ablate stellate ganglia neuron activity in hypertension.
