# Supplementary figures and images for: Downregulation of M Current Is Coupled to Membrane Excitability in Sympathetic Neurons Before the Onset of Hypertension
Source: Hypertension. 2020 Oct 12;76(6):1915–23. doi: 10.1161/HYPERTENSIONAHA.120.15922 (PMC8360673; doi:10.1161/HYPERTENSIONAHA.120.15922)

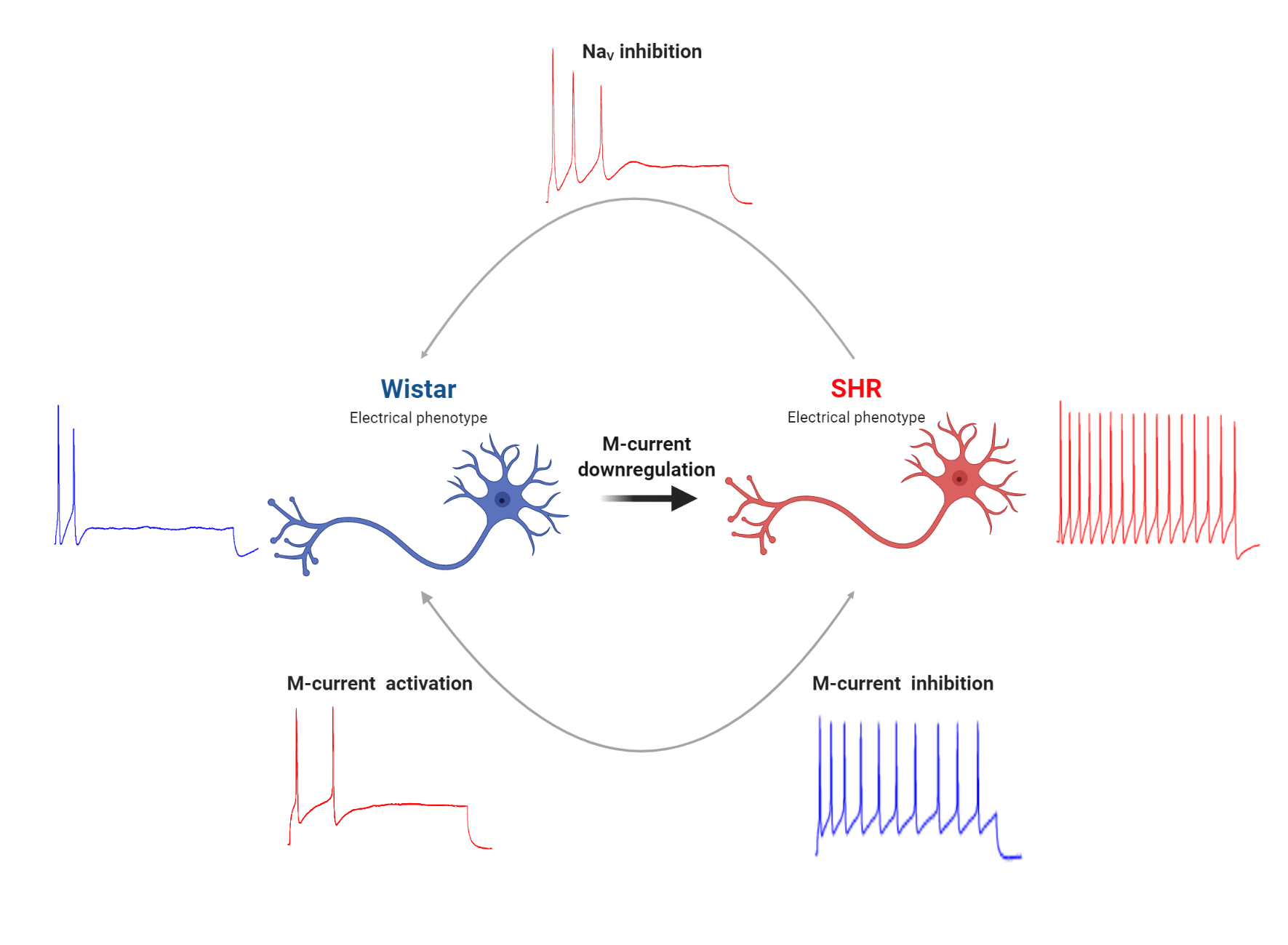

Supplement: Supplementary file 1 [file hyp-76-1915-s001.jpg]
