# Supplementary material for: Downregulation of M Current Is Coupled to Membrane Excitability in Sympathetic Neurons Before the Onset of Hypertension
Source: Hypertension. 2020 Oct 12;76(6):1915–23. doi: 10.1161/HYPERTENSIONAHA.120.15922 (PMC8360673; doi:10.1161/HYPERTENSIONAHA.120.15922)
Supplement: Supplementary file 2 [file hyp-76-1915-s002.pdf]

Online supplement

Downregulation of M current is coupled to membrane excitability in sympathetic neurons before the onset of hypertension

Harvey Davis, Neil Herring, David J Paterson

Correspondence: Harvey Davis, University of Oxford, harvey.davis@dpag.ox.ac.uk, 07456676414 **or** David J Paterson, University of Oxford, david.paterson@dpag.ox.ac.uk, 01865 272430

Supplementary methods

Cell isolation and culturing Stellate ganglia were dissected and immediately transferred to ice-cold HEPES buffered L15 media (L1518, Thermofisher, US). Ganglia were then cut into 2 mm sections using surgical scissors, and enzymatically digested at 37 ^{o}C first using 1 mg/ml Collagenase IV (Worthington, US) in L15 for 25 minutes, followed by 30 minutes in 2 mg/ml Trypsin (Worthington, US) in Ca^{2+} and Mg^{2+} free Hanks buffered salt solution (Thermofisher, US). Enzymes were then inhibited using two washes of a blocking solution containing 10% FBS. The tissue was then suspended in a plating media containing Neurobasal plus media, B27 plus, 100 ng/ml 2.5s NGF, 25 mM glutamax and 50 units/ml Pen-strep and mechanically disrupted using a fire-blown glass pipette. The cell suspension was then plated onto Poly-D-lysine coated Fluorodish 35 mm dishes (WPI, US), which had been previously incubated for 2 hours with 1 ug/ml laminin, a concentration chosen to allow cell adhesion and survival, but limiting neurite outgrowth [43]. The cells were then incubated at 37 ^{o}C with 5% CO_2 for a period of 1-5 days in vitro before use. All datasets were recorded from at least 2 cultures, with each culture requiring 4 animals. Phase contrast microscopy was used to enable neuronal identification. Neurons were identified in dissociated culture based on their large size and circular somata relative to the surrounding cell types. Each culture was produced from a minimum of 2 animals, with each experiment being performed on a minimum of two cultures.

Electrophysiological Data acquisition All electrophysiological data were acquired using Winwcp (Version 5.4.0) and recorded via a Multiclamp 700B amplifier (Molecular Devices, US) with an axon digidata 1550A (Molecular devices, US) digitizer. All current clamp recordings were sampled at 10 kHz. M-current deactivation curves were sampled at 10 KHz.

Perfusion Cells were constantly perfused at a rate of 5-6 ml/min, drugs were applied via this perfusion system. Most drugs were continuously applied for a duration of 5 minutes before data acquisition, XE-991 and substituted LiCl were perfused for 10 minutes before recording. Recordings were performed at room temperature.

Perforated patch-clamp recordings Amphotericin B (0.48 mg/ml) was used as the perforating agent [44], recordings were initiated after low series-resistance electrical access was achieved (<30 MΩ) and stable for a period of five minutes. All voltage clamp recordings used 70% series resistance compensation and capacitance cancellation. Current clamp recordings were bridge balanced. All recordings were monitored throughout, and recordings with R_S changes > 20% were discarded. The external recording solution for both current clamp and voltage clamp recordings was as follows: 5.2 mM KCl, 140 mM NaCl, 1 mM $MgCl_2$, 1.8 mM $CaCl_2$, 10 mM HEPES, 10 mM D-Glucose. External solution pH was adjusted to 7.4 with NaOH. For removal of external calcium, calcium was excluded from this solution and 5 mM EGTA was added. The internal solution was composed as follows: 145 mM K^+-Aspartate, 2.2 mM EGTA, 10 mM HEPES, 1 mM $MgCl_2$. Internal pH was adjusted to 7.3 using KOH.

Whole cell patch-clamp recordings Whole cell action potential recordings with Rs values >12 MΩ discarded. Current clamp recordings were bridge balanced and membrane potentials were corrected for liquid junction potentials. All recordings were monitored throughout, and recordings with R_S changes > 20% were discarded. Single action potentials were evoked via a 10ms positive current injection, at the minimal required injection size. External solution composition was the same as for perforated patch. For LiCl substitution experiments, 140 mM LiCl was substituted for NaCl. The internal solution was as follows: 130 mM K^+-Gluconate, 10

mM KCl, 10 mM HEPES, 10 mM Na^+-Phosphocreatine, 4 mM MgATP, 0.3 mM Na_2GTP. Internal pH was adjusted to 7.3 with KOH. To study K_{Na}, NaCl was substituted for LiCl to allow an inward g_{Li} via Na_V channels but prevent activation of slick and slack channels by intracellular sodium [45]. For Ca^{2+} free experiments, Ca^{2+} was removed from the external solution and 5 mM EGTA, a Ca^{2+} buffer, was added.

qRT-PCR Total RNA from whole flash frozen stellate ganglia was isolated using a RNeasy minikit (Qiagen, US) and immediately stored on dry ice before cDNA library preparation. For cDNA synthesis, Superscript IV VILO with ezDNase genomic DNA depletion (Thermofisher, US) was used, cDNA was then stored at -80°C until required. Taqman PCR primers were used for the transcript identification of KCNQ2 (Rn00591249_m1), KCNQ3 (Rn00580995_m1), KCNQ5 (Rn01512013_m1), SCN10A (Rn00568393_m1), where either GAPDH (Rn01775763_g1) or B2M (Rn00560865_m1) were used to normalize values to their age matched controls via the ΔΔCT method [46]. Samples were measured on an ABI Prism 7000 (Thermofisher, US) as per the standard protocol for taqman. The known clinical characteristics of the 4 donor human stellate ganglia samples were as follows: Sample ID 19, Male, 77 years old, 30-35% left ventricular ejection fraction, nonischemic cardiomyopathy and ventricular fibrillation; Sample ID 20, Female, 61 years old, Hypertension and hypothyroidism; Sample ID 23, Male, 19 years old, left ventricular ejection fraction 55-60%, normal cardiac function; Sample ID 24, Male, 62 years old, left ventricular ejection fraction 50%, polymorphic ventricular tachycardia.

Cryosectioning and Immunohistochemistry Freshly isolated stellate ganglia were immediately transferred to 4% paraformaldehyde for 1-2 hours, after which the tissue was incubated overnight in 20% sucrose-PBS at 4 °C, before embedding in OCT compound (Tissue-Tek). Tissue was then frozen and stored at -80 °C until cryosectioning the tissue as 12 µm sections. Slides were then permeabilized in 0.3% triton-X for 30 minutes at room temperature, before blocking for 2 hours in 1% BSA, 5% donkey serum. Sections were then incubated for 24 hours with primary antibodies at 4°C, followed by five 5 minute washes in PBS and 2 hours incubation with the relevant secondary antibodies. Sections were subsequently washed 3 times in PBS, and incubated with DAPI/PBS for 5 minutes, before a final 2 washes in PBS. Slides were then mounted with 50% glycerol in PBS before imaging. Sections were imaged on a Zeiss LSM 880 Airy Scan Upright laser-scanning confocal microscope with a Plan-Apochromat 20x/0.8 M27 objective. Sections were DAPI stained, labelled with a mouse anti-TH antibody (66334-1-Ig) (ProteinTech, US) and a rabbit antibody against either KCNQ2 (ab22897), KCNQ3 (ab66640) or KCNQ5 (ab66740) (Abcam, UK). For secondary antibodies, 1:200 Donkey anti-mouse Alexa Fluor 555 (A-31570) and 1:200 Donkey anti-rat Alexa Fluor 488 (A-21208) were used (Invitrogen).

Single cell RNA-sequencing A pooled single cell suspension of stellate ganglia cells from six animals per strain was prepared via enzymatic dissociation as described under cell culture methods. Following blockade of enzymatic activity via three washes in blocking solution, the cell solution was transferred to phosphate buffered saline. The cell solutions were immediately transferred to ice and transported to the Wellcome Trust Centre for Human Genetics (WTCHG) for scRNAseq via 10x genomics chromium (10x genomics, US) (Single Cell 3' v3) and Illumina hiseq 4000 (Illumina, US). This approach achieved 66-72K mean reads per cell and a sequencing depth of 53-55% per cell before filtering.

Initial analysis was performed by the WTHCG using the cell ranger pipeline (x10 genomics, US) (Cell ranger, v3.0.2) (Rnor6.0) with default parameters, before the data were exported to

Seurat (v3.0) [47] and analyzed in house. Cells were excluded in Seurat if the number of counts per cell was less than 4000 or percentage of mitochondrial genes was equal to or less than 0.3. For FindVariableFeatures we used 10000 features and the election method VST. Data was intergrated using 30 dimensions, 30 principle components were using for PCA analysis. UMAP and TSNE, FindNeighbours were ran with 19 dimensions. Findclusters was ran with a resolution of 0.6. Differential expression analysis was performed via MAST [48] within Seurat. To determine multiple neuron populations for supplementary figure 5, findclusters was ran at a resolution of 10, and neuron-like groups were subset for further analysis. Individual groups were then visually identified based upon clear separation from neighbouring subgroups.

Electrophysiological Data analysis Analysis of firing rate data were performed in WinWCP (v5.4.0). M-current deactivation curves were analyzed within Clampfit (v10.7, Molecular Devices, US). Graphs were produced in either Graphpad prism (v8.2.1) or ggplot2 (v3.2.1) and Waffle (v1.0.1) in R (Version 3.5.3). Statistical analysis was performed in GraphPad prism, all relevant datasets were normality tested.

Firing rate was taken as the maximum firing rate elicited by a range of 10 pA current injections between 10-200 pA. Membrane potential was monitored for stability during drug wash in and cells with large jumps in membrane potential were discarded.

Action potential parameters were measured from the first sequential 50 pA current step that induced an action potential. Peak amplitude (mV) was taken as the difference between the average baseline and maximum peak response of the action potential. Action potential upstroke (mV/ms) was taken as the maximum velocity from baseline to the peak amplitude.

Input resistance was calculated based upon a series of hyperpolarizing and depolarizing current injections ranging from -200 to 200 pA in amplitude in 10 pA increments [49]. The average value of the final 100 ms was analyzed. As previously classified, small hyperpolarizing pulses were assumed to elicit the least active processes and any points that departed from linearity with these points or contained visible active processes in the final 200 mS of current injection were excluded.

Liquid junction potentials were calculated in JPCalcW [50] in Clampex (v11.0.3) (Molecular Devices, US), where ion availabilities were used instead of concentrations. Free Ca^{2+}, ATP, EGTA and Mg^{2+} for internal solutions were estimated via MaxChelator (v8)[51] when relevant. For perforated-patch voltage-clamp and current clamp recordings a Liquid junction potential of 24.3 mV was calculated, without correction for the perforated patch Donnan potential [52]. Whole cell current clamp recordings had an estimated liquid junction potential of -15.7 mV.

Supplementary Figures

Figure S1 Analysis of the relationship between cell capacitance, days in vitro (DIV) and maximum firing rate in either strain as measured by perforated patch clamp. (A) The number of days in vitro does not affect the maximum neuronal firing rate in Wistar neurons (Median) (DIV1, 2 Hz, n = 19; DIV2, 2 Hz, n = 14; DIV3, 1.5 Hz, n = 28; DIV4, 1 Hz, n = 5) (Kruskal-Wallis test, n = 66, p = 0.48). (B) There is no clear relationship between cell capacitance and firing rate in Wistar neurons. (C) The number of days in vitro does not affect the maximum neuronal firing rate in SHR neurons (Median) (DIV1, 5.5 Hz, n = 18; DIV2, 9 Hz, n = 22; DIV3, 9.5 Hz, n = 18; DIV4, 4 Hz, n = 10; DIV5, 8.5 Hz, n = 2) (Kruskal-Wallis test, n = 70, p = 0.35). (D) There is no clear relationship between cell capacitance and firing rate in SHR neurons. (E) There is not a significant difference in the number of days in vitro between strains (Median) (Wistar, 2.5 days, n = 66; SHR, 2 days, n = 70) (Mann-Whitney test, p = 0.83). (F) There is no significant difference in capacitance between strains, although there is a non-significant trend (Median) (Wistar, 26.74 pF, n = 66; SHR, 23.35 pF, n = 70) (Mann-Whitney, p = 0.27). (G) Dependence of firing rate on membrane potential was determined in SHR neurons by applying a pre-pulse in the range -10 to -100 pA for 1 second before applying a 150 pA positive current injection to elicit cell firing. No difference was found (Friedman test, n = 26, p = 0.165).

Figure S2 Single cell RNA-sequencing and a panel of pharmacological inhibitors were used to determine remaining channels involved in SHR enhanced firing, that may be targetable for the reduction of aberrant sympathetic hyperactivity or key to the SHR sympathetic pathology. Single-cell RNA-sequencing was used to identify the cell specific expression patterns of a range of channel subunits which are typically implicated in the control of firing rate in other neuronal populations. Here percentage expressed indicates the number of cells per cluster identity exhibiting transcript expression and average expression refers to the average expression per cluster identity. The table highlights the effect of selective inhibitors on firing rate for channels not shown in the main manuscript. Data shown for 1s 50 pA, 100 pA, 150 pA current injections and at maximum firing frequency between 0-200 pA current amplitude. Paired comparisons were made by Wilcoxon tests.

Figure S3 Immunohistochemistry showing M-current subunit protein in TH-positive neurons in cryosections of 5-6 week old Wistar stellate ganglia (KCNQ2, KCNQ3 and KCNQ5).

Figure S4 Neural subtypes correlate with measures of I_M and I_{Na}. (A) A summary figure for proposed contribution of M-current and I_{Na} to firing rate classes in stellate ganglia neurons. (B) M-current density was lower in Tonic firing neurons than phasic 2 neurons (Median±IQR) (Phasic 1, -4.09 pA/pF, n=7; Phasic 2, -7.18 pA/pF, n=12; Tonic, -2.62 pA/pF, n=18) (Kruskal-Wallis test; p = 0.0019) (Dunn's multiple comparisons test; Phasic 2 vs Tonic, p = 0.0012). (C) Upstroke velocity for phasic 1, phasic 2 and tonic neurons revealed a higher Tonic velocity (Median ± IQR) (Phasic 1, 34.71 mV/ms, n = 20; Phasic 2, 63.65 mV/ms, n = 32; Tonic, 85.38 mV/ms, n = 37) (Kruskal-Wallis test; p = 0.0018) (Dunn's multiple comparisons test; Phasic 1 vs Tonic, p = 0.0012). (D) Action potential amplitude was significantly higher for phasic 2 and tonic neurons than in phasic 1 neurons (Mean ± SEM) (Phasic 1, 68.30 ± 4.49 mV, n = 21; Phasic 2, 78.25 ± 2.25 mV, n = 32; Tonic, 81.96 ± 2.14mV, n = 37) (One-way ANOVA; p = 0.0071) (Holm-sidak's multiple comparisons test; Phasic 1 vs Phasic 2, p = 0.049; Phasic 1 vs Tonic, p = 0.0054).

Figure S5 Experimental demonstration of the role of I_{Na} and I_M in determining firing class. Nav1.6 inhibition significantly reduced firing rate in M-current (3 µM XE-991) inhibited Wistar neurons (Median) (Control, 2 Hz; XE-991 treated, 3 Hz; XE-991 and 4,9-Anhydrotetrotoxin treated, 1 Hz) (Friedman test, n = 10, p = 0.0084) (Dunn's Multiple comparisons test; XE-991 treated vs XE-991 + 4,9-Anhydrotetrotoxin, p = 0.038).

Figure S6 Evidence of multiple populations of sympathetic neurons. (A) Visually identified cell clusters of potential sympathetic neurons. (B) Expression of key transcripts for noradrenaline synthesis and breakdown are shown for each neuron cluster. Of these groups only Neurons 1 and Neurons 2 appear to adequately express the full noradrenaline synthesis pathway. (C) Sympathetic Neurons 1 are shown to be have low expression of CHRM2 and NPY, two physiologically important genes, in contrast to high expression in Sympathetic Neurons 2. For reference, these groups are termed Type A sympathetic neurons (Sympathetic Neurons 1), and type B sympathetic neurons (Sympathetic Neurons 2). Importantly, we also demonstrate that both groups have similar ion channel profiles, with no significant differences observed between groups.

Figure S7 Top changes in neuronal cluster gene expression between Wistar and SHR strains (relative to Wistar) as determined by Single cell RNA-sequencing. (A) Top 50 genes that are significantly increased in the SHR. (B) Top 50 genes that are significantly decreased in the SHR. (C) Top 50 genes expressed independent of strain in Wistar and SHR neurons. (D) Channel encoding genes assessed for comparison of genes involved in firing rate.

Figure S8 Unbiased markers for major cell clusters found in the stellate ganglia as detected by Seurat analysis. (A) Top 30 markers for the immune cell cluster. (B) Top 30 markers for the Vascular smooth muscle cell cluster. (C) Top 30 markers for the glial cell cluster. (D) Top 30 markers for the Fibroblast cell cluster. (E) Top 30 markers for the endothelial cell cluster. (F) Top 30 markers for sympathetic neuron clusters.

S1

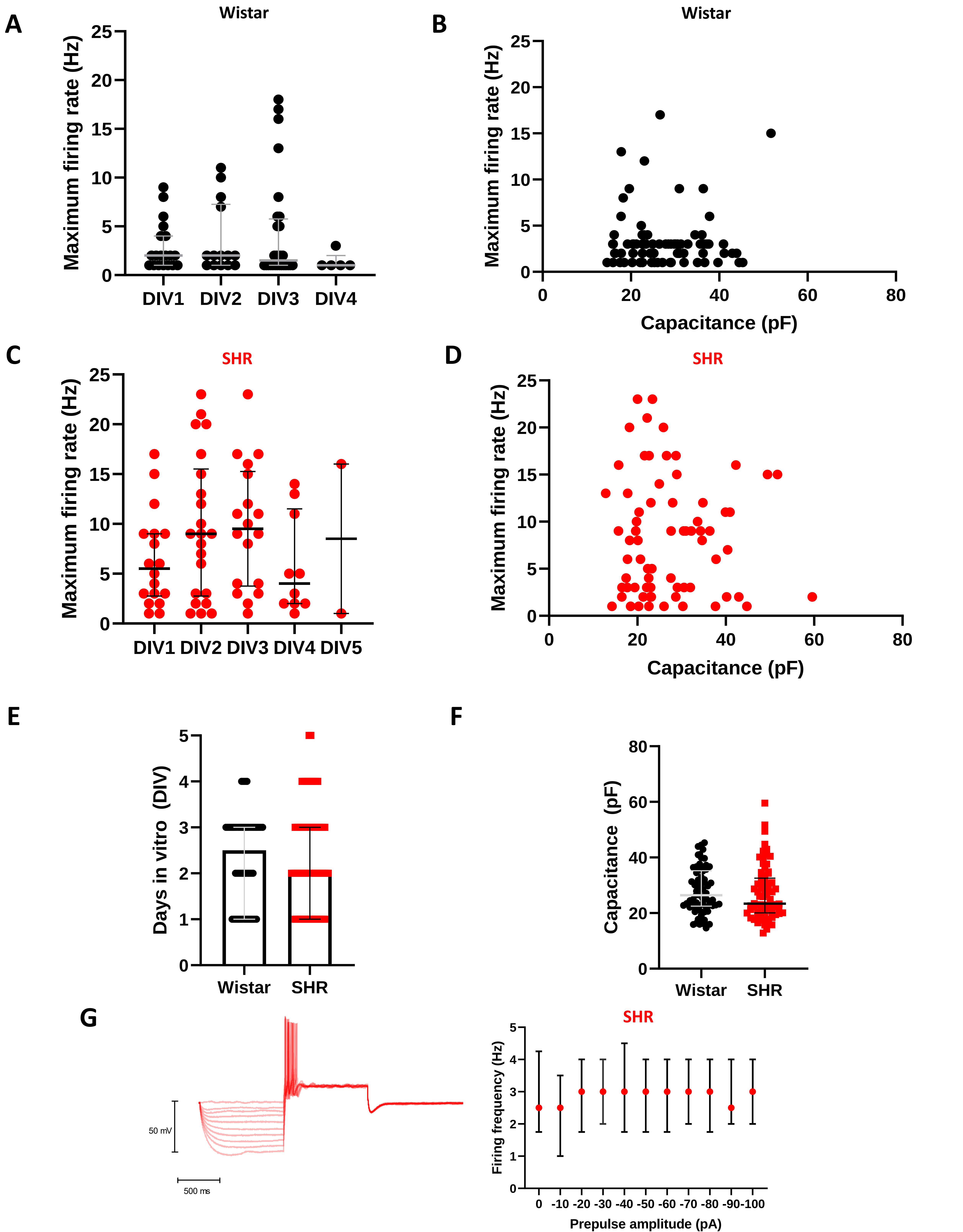

A
Wistar
Maximum firing rate (Hz)
0
5
10
15
20
25
DIV1
DIV2
DIV3
DIV4
B
Wistar
Maximum firing rate (Hz)
Capacitance (pF)
0
20
40
60
80
C
SHR
Maximum firing rate (Hz)
DIV1 DIV2 DIV3 DIV4 DIV5
D
SHR
Maximum firing rate (Hz)
Capacitance (pF)
E
Days in vitro (DIV)
Wistar
SHR
F
Capacitance (pF)
Wistar
SHR
G
50 mV
500 ms
SHR
Firing frequency (Hz)
0 -10 -20 -30 -40 -50 -60 -70 -80 -90 -100
Prepulse amplitude (pA)

S2

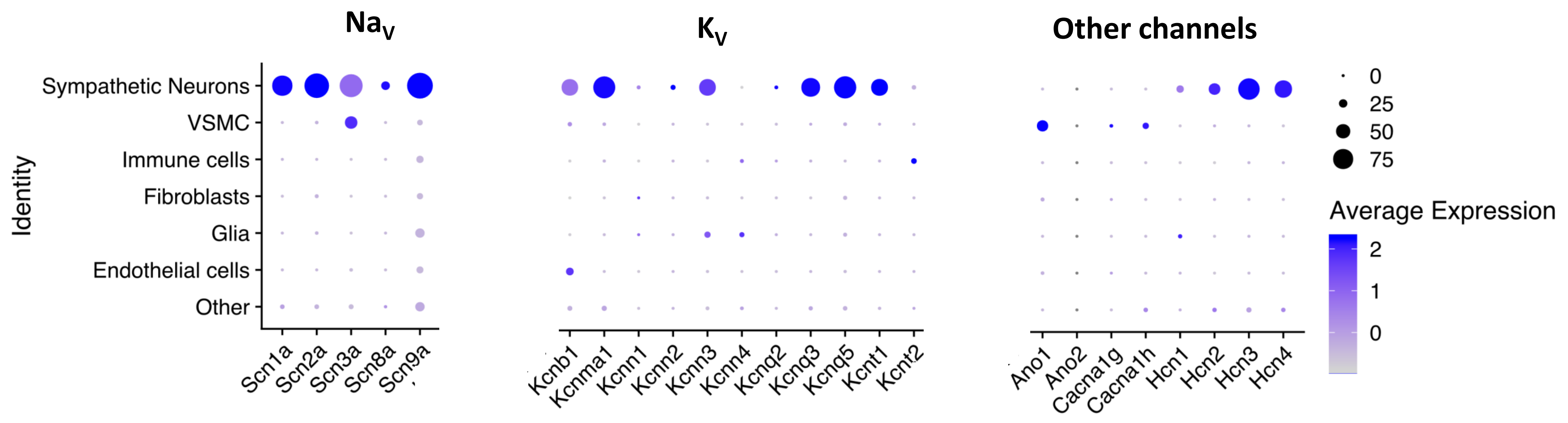

	HCN		T-type		Ca2+ free	SK	BK	CaCC	Nav1.7		K_v2.1	KNa
**Drug**	Ivabradine	ZD7288	TTA-A2	TTA-P2	EGTA	Apamin	Iberiotoxin	Ani9	Protx-III	Huwentoxin-IV	Guangxitoxin	Lithium
**Concentration**	3µM	1µM	500nm	1µM	5mM	200nM	100nM	1µM	50nM	50nM	100nM	N/A
**Perforating agent**	Amphotericin	Amphotericin	Amphotericin	Amphotericin	Amphotericin	Amphotericin	Amphotericin	Amphotericin	Amphotericin	Amphotericin	Amphotericin	Whole cell
**Δ Firing rate 150pA**	-1	0	0	-1	-1	1.5	0	0.5	0	2.5	**3**	0
P value	*0.67*	*0.5*	*>0.99*	*0.60*	*0.59*	*0.9375*	*0.81*	*0.87*	*0.38*	*0.11*	***0.0078***	*0.84*
**Δ Firing rate 100pA**	0	0.5	0	-0.5	1	1	0	0.5	0	0.5	**2.5**	0
P value	*0.75*	*0.95*	*0.25*	*0.89*	*0.22*	*0.0625*	*0.94*	*0.55*	*0.44*	*0.19*	***0.0156***	*0.5*
**Δ Firing rate 50pA**	0	0.5	1	0	0	3	0	0.5	-0.5	0	0	0
P value	*0.19*	*0.25*	*0.16*	*>0.99*	*0.094*	*0.0938*	*>0.9999*	*0.5156*	*0.63*	*0.5*	*>0.9999*	*0.25*
**Δ Maximum firing rate**	0	0	0	-0.5	0.5	**4**	0	1	-0.5	1	**1.5**	0
P Value	*0.13*	*>0.99*	*0.63*	*0.51*	*0.61*	***0.0156***	*0.60*	*0.29*	*0.67*	*0.19*	***0.0391***	*0.81*
**n**	10	8	9	10	12	8	12	10	8	8	8	10

S3

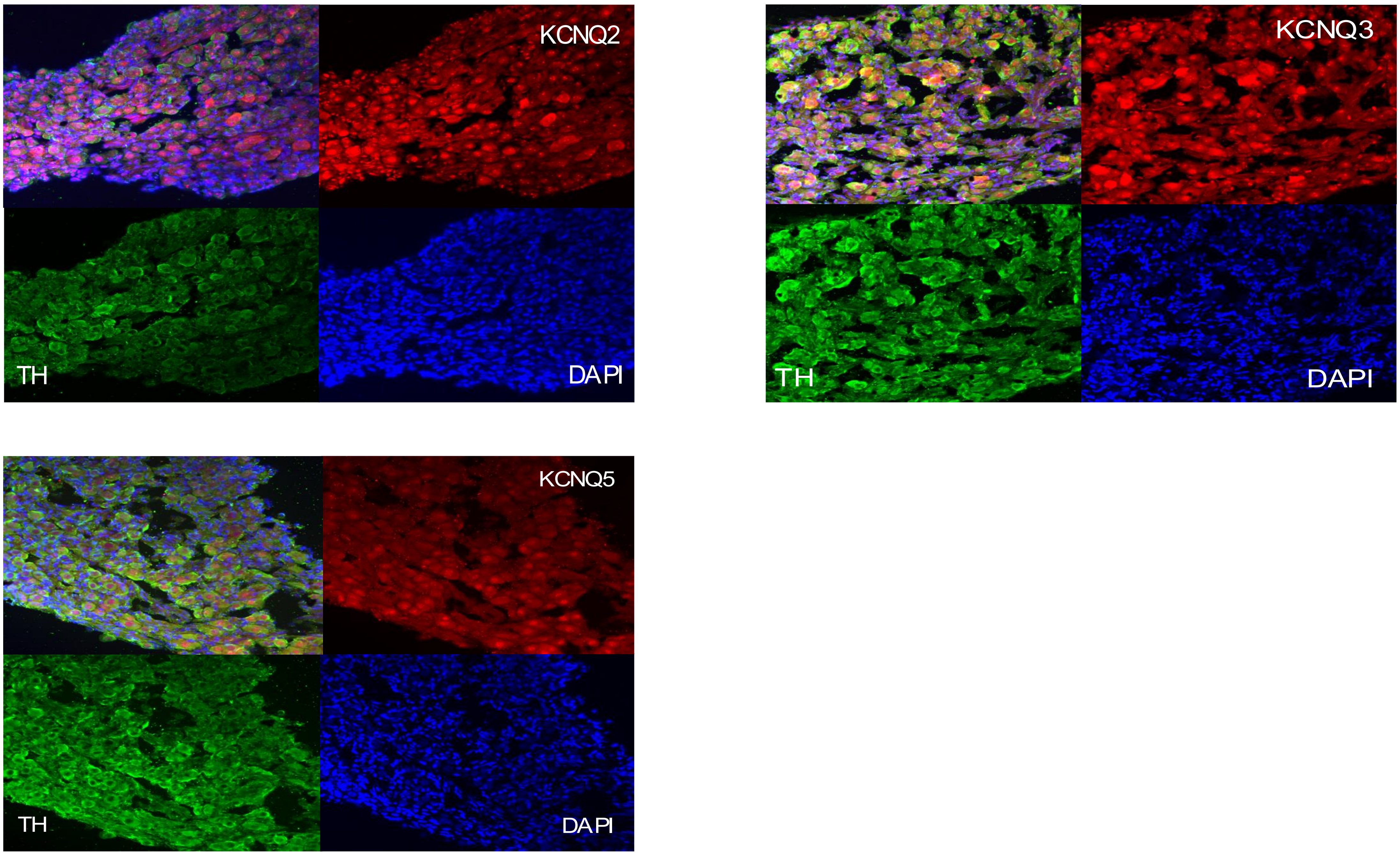

S3

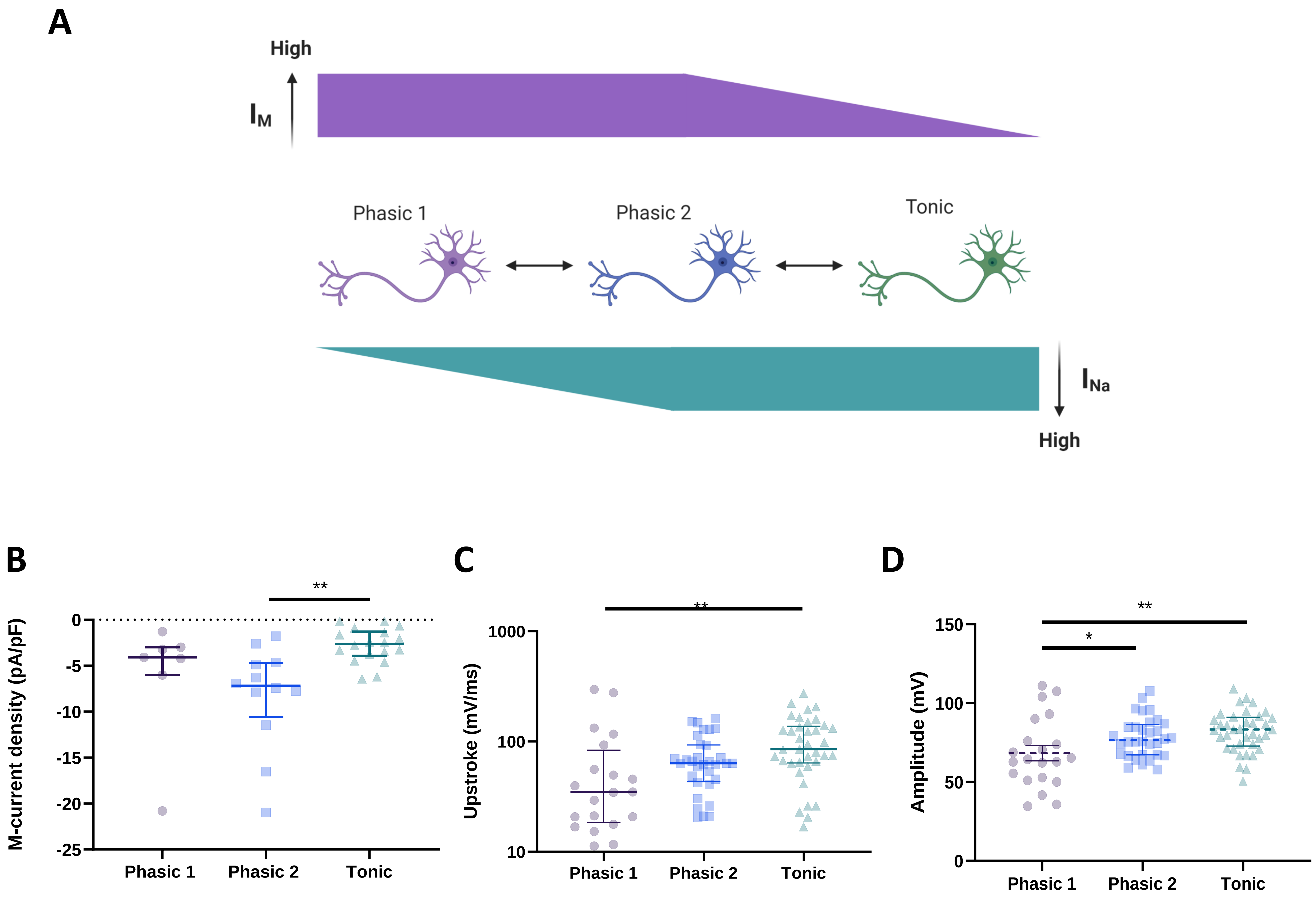
A
High
I_M
Phasic 1
Phasic 2
Tonic
I_{Na}
High
B
**
M-current density (pA/pF)
0
-5
-10
-15
-20
-25
Phasic 1
Phasic 2
Tonic
C
**
Upstroke (mV/ms)
1000
100
10
Phasic 1
Phasic 2
Tonic
D
**
*
Amplitude (mV)
150
100
50
0
Phasic 1
Phasic 2
Tonic

S4

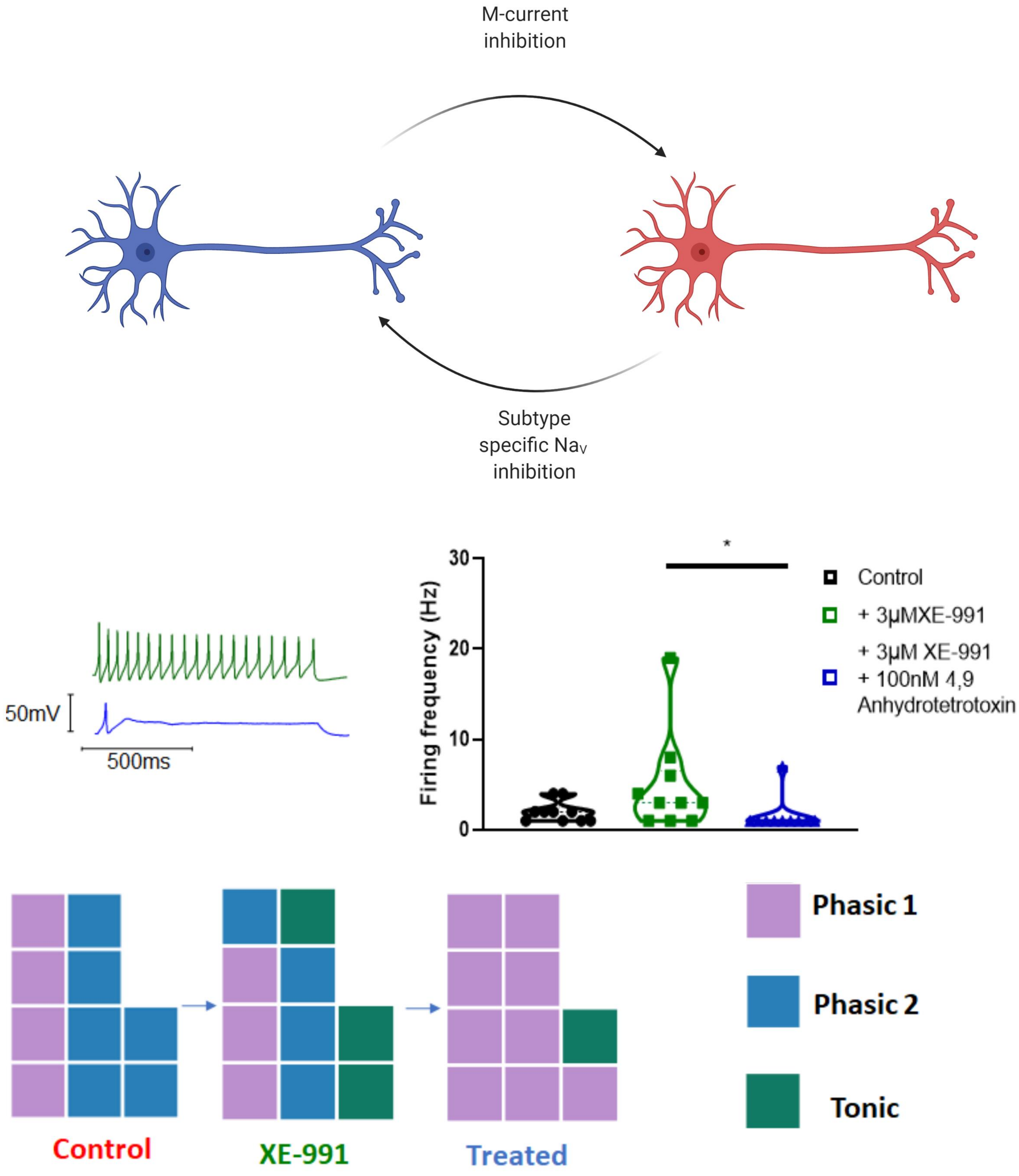
M-current
inhibition
Subtype
specific NaV
inhibition
50mV
500ms
Firing frequency (Hz)
30
20
10
0
*
Control
+ 3μMXE-991
+ 3μM XE-991
+ 100nM 4,9
Anhydrotetrotoxin
Phasic 1
Phasic 2
Tonic
Control
XE-991
Treated

S5

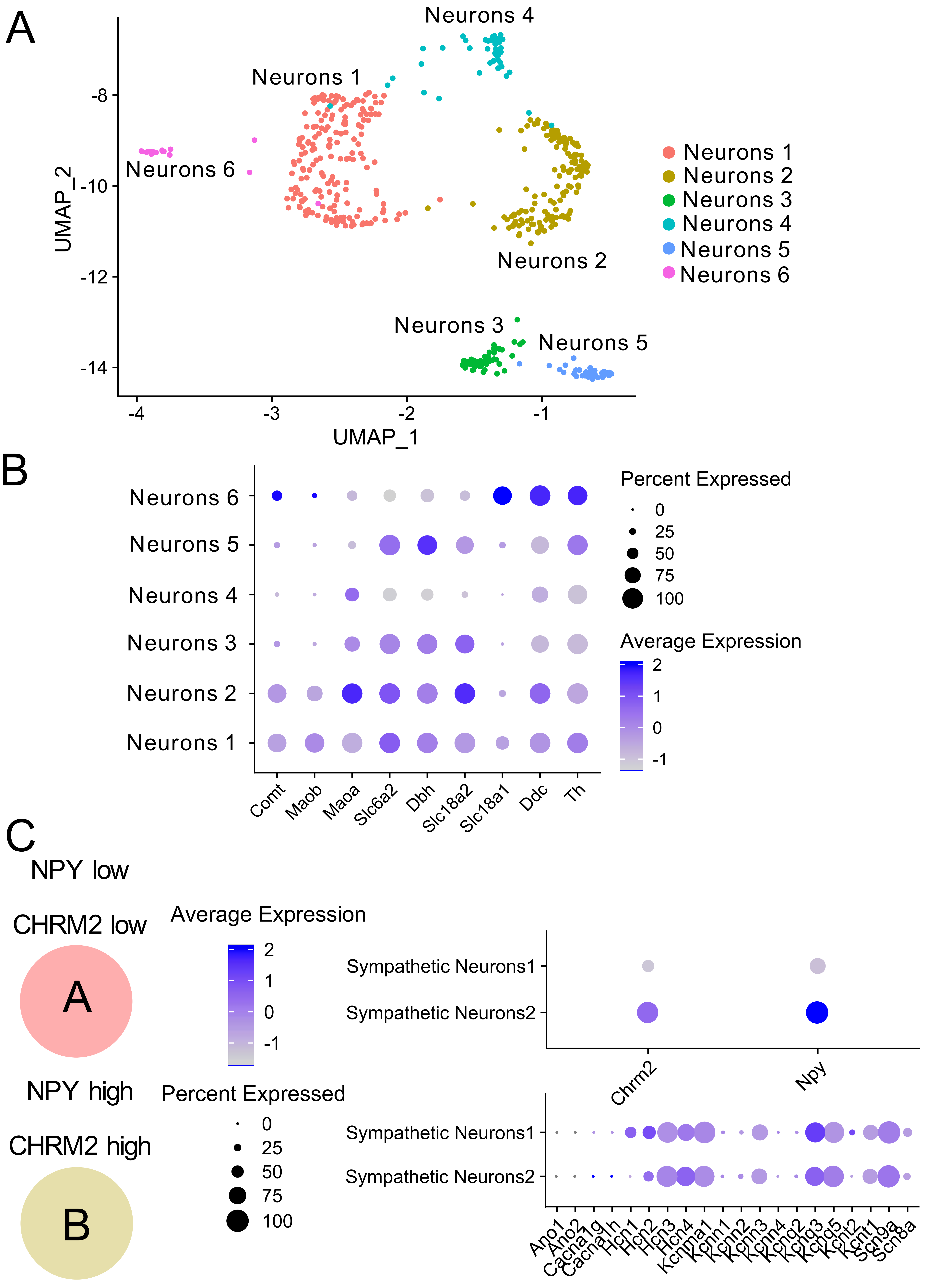

A
Neurons 1
Neurons 2
Neurons 3
Neurons 4
Neurons 5
Neurons 6
UMAP_1
UMAP_2
-8
-10
-12
-14
-4
-3
-2
-1
B
Percent Expressed
0
25
50
75
100
Average Expression
2
1
0
-1
Comt
Maob
Maoa
Slc6a2
Dbh
Slc18a2
Slc18a1
Ddc
Th
C
NPY low
CHRM2 low
A
NPY high
CHRM2 high
B
Average Expression
Percent Expressed
Sympathetic Neurons1
Sympathetic Neurons2
Chrm2
Npy
Ano1
Ano2
Cacna1g
Cacna1h
Hcn1
Hcn2
Hcn3
Hcn4
Kcnma1
Kcnn1
Kcnn2
Kcnn3
Kcnn4
Kcnq2
Kcnq3
Kcnq5
Kcnt2
Kcnt1
Scn9a
Scn8a

S6

Neurons

A

Increased expression

Gene	Log2Fold change	Adjusted P value
Jund	1.5082516	4.367859e-101
S100a6	1.0578275	2.450488e-09
LOC100364435	0.7454090	5.585682e-14
Lgals3	0.7439313	1.889339e-21
Rgs10	0.6371928	2.374208e-20
Cox7c	0.6360328	1.545634e-14
Ifi27l2b	0.5969734	1.413566e-10
Rps25	0.5790300	2.864862e-22
Nbl1	0.5747978	6.333336e-11
Lgals1	0.5714637	1.392294e-13
RT1-A1	0.5460656	7.680405e-20
Atp5f1e	0.5318127	1.876125e-08
Tmsb4x	0.5265573	3.358830e-05
Lix1	0.5142321	3.978586e-15
Cops9	0.4927932	1.323195e-15
Gsta4	0.4855133	3.403365e-50
Gabarap	0.4794821	8.050229e-08
Sncg	0.4733403	9.515786e-06
Lynx1	0.4652957	2.303662e-13
Fxyd6	0.4546705	1.184943e-11
Gap43	0.4534140	1.044454e-11
LOC100359583	0.4523299	6.131520e-09
Ube2s	0.4504232	7.396337e-23
LOC103689961	0.4501770	5.011713e-04
Rps15a	0.4469772	3.038277e-04
Nrsn1	0.4431640	3.459381e-19
Cd24	0.4429852	4.084656e-10
Pfn1	0.4382201	5.952396e-06
Clic3	0.4381656	2.269674e-21
Id1	0.4362874	3.333847e-09
S100a11	0.4360989	3.040743e-07
Cstb	0.4340093	1.223860e-12
Dynll1	0.4320592	6.394903e-09
Rpl6	0.4313981	2.959553e-07
Mt3	0.4196665	1.086267e-03
Ywhah	0.4167402	9.640197e-09
S100a10	0.4156335	5.061056e-08
Crip1	0.4131412	1.000000e+00
Cox6c	0.4121257	2.138002e-05
RGD1564664	0.4105464	6.250173e-09
Cox8a	0.4105403	2.700923e-03
Ctxn1	0.4092833	2.431107e-07
Tppp3	0.4087822	1.062926e-12
Fabp5	0.4030572	4.743981e-08
Atp5mc2	0.4022625	1.413702e-05
Rps27a.1	0.4015203	1.378063e-02
Uqcrq	0.3967584	1.291659e-07
Ndufb2	0.3958326	9.469155e-06
LOC687780	0.3952969	4.839876e-04
Sncb	0.3943067	5.081632e-09
Rps4x.1	0.3931921	1.000000e+00
Ost4	0.3926721	8.414767e-07
Fkbp1b	0.3924926	1.496808e-04
Rpl41	0.3922107	9.844968e-04
Rpl24	0.3918698	5.646911e-02
Elob	0.3912504	9.582211e-06
Rpl10	0.3908021	9.266765e-06
Id3	0.3898860	2.287913e-09
Timm8b	0.3896933	1.247257e-06
Atp5pf	0.3882082	1.176928e-06

B

Decreased expression

Gene	Log2Fold change	Adjusted P value
AC134224.1	-1.1919235	7.390003e-14
AC134224.3	-1.1243569	2.337894e-01
Rsrp1	-1.0110946	8.436000e-07
Cd9	-1.0105276	2.551646e-14
Pcp4	-0.8881339	2.445960e-21
Avil	-0.8147849	1.833540e-32
Apoe	-0.8061488	1.826096e-01
Snhg11	-0.7870100	1.000000e+00
Clasrp	-0.7413731	4.021941e-03
Gria2	-0.7367756	2.002588e-10
Snrnp70	-0.7127888	4.602979e-02
AABR07043389.1	-0.7011961	7.905533e-25
Rock1	-0.6985003	1.892195e-04
Tnpo1	-0.6799992	1.659938e-15
Mcf2l	-0.6690244	1.769559e-07
Slc12a3	-0.6554360	2.632086e-24
Xkr6	-0.6535802	3.527137e-01
Insrr	-0.6426622	6.661967e-04
AC134224.2	-0.6362384	1.233835e-04
Kifc2	-0.6291232	3.462901e-03
Alcam	-0.6282179	2.411339e-07
Macf1	-0.6187952	1.524047e-14
Ddc	-0.5950884	2.365693e-18
Rbfox1	-0.5945057	5.243143e-03
Pnisr	-0.5908631	6.034754e-03
Leng8	-0.5850844	1.022591e-03
Aqp1	-0.5821055	4.037932e-08
Ssbp4	-0.5752207	2.196309e-01
Abca7	-0.5728495	2.284495e-03
Epha5	-0.5728401	8.027044e-07
Ogt	-0.5657906	7.585591e-07
Srrm2	-0.5603236	1.537572e-02
Srsf2	-0.5566520	3.052943e-04
Zbtb20	-0.5551126	3.212383e-05
Stk38	-0.5509250	1.375099e-06
Carmil3	-0.5479098	4.352376e-03
Lss	-0.5475808	1.238291e-03
Agrn	-0.5446013	3.798112e-04
Prkg2	-0.5419457	9.840020e-18
Ddx39b	-0.5372624	6.367377e-05
Brinp2	-0.5350721	4.241865e-05
Pnn	-0.5290578	6.977921e-02
Pcmtd2	-0.5265765	3.677216e-03
Sparc	-0.5206209	1.000000e+00
Pabpn1	-0.5186682	9.728370e-01
Vav2	-0.5151115	2.180735e-06
Pclo	-0.5125366	3.626956e-08
Arhgap21	-0.5025987	7.737147e-11
P3h3	-0.5017257	5.764083e-10
Trafd1	-0.4986911	4.079081e-03
Zcchc7	-0.4942523	1.663439e-01
Nfat5	-0.4886901	4.319940e-05
Atp13a2	-0.4856006	2.457154e-08
Taf1d	-0.4822939	1.000000e+00
Luc7l3	-0.4804302	3.571888e-03
Atxn2l	-0.4792462	6.461217e-02
Cacna1b	-0.4773798	1.671714e-04
Chl1	-0.4741254	9.661983e-10
Abca8a	-0.4734990	1.000000e+00
RT1-CE4	-0.4724680	3.738463e-21

C

Top expressed

Gene	Wistar Reads
Mt-cyb	5.441520
Mt-co1	5.067902
Mt-nd2	4.896349
Mt-nd1	4.574465
Mt-nd4	4.544456
Tuba1a	4.350063
LOC100364435	4.156522
Prph	4.076758
AC134224.1	3.993742
Stmn2	3.834467
Tubb3	3.824851
Map1b	3.812369
Sncg	3.809325
Tmsb4x	3.784265
Hsp90ab1	3.410114
Snhg11	3.294435
Atp6v0c	3.286958
Uchl1	3.257605
Atp1a1	3.224183
Actg1	3.214234
Syt1	3.194060
Tubb5	3.174731
Rtn1	3.150431
Stmn3	3.148980
Tubb2a	3.106304
Fth1	3.086398
Atp1b1	3.023032
Tubb2b	3.013394
Nefl	3.004773
Snap25	2.914318
S100a6	2.890331
Zwint	2.872957
Syt4	2.869539
Stmn1	2.865590
Ndrg4	2.860553
Cd9	2.846434
Elavl2	2.840360
Ndfip1	2.839060
Slc6a2	2.825227
Basp1	2.824477
Rtn3	2.799926
Calm1	2.788565
Ywhah	2.779392
Reep5	2.768612
Cyb561	2.765191
Ntrk1	2.736131
Dst	2.726988
Tspan8	2.718403
Cfl1	2.716252
App	2.709063
Cd24	2.707676
Nsg1	2.702262
LOC103692716	2.695446
Rtn4	2.687459
Gap43	2.659190
Bcat1	2.658582
LOC108348172.1	2.656803
Aldoa	2.631778
Lgals1	2.622184

D

Channels assessed

Gene	Channel
Scn1a	Na_v1.1
Scn2a	Na_v1.2
Scn3a	Na_v1.3
Scn4a	Na_v1.4
Scn5a	Na_v1.5
Scn7a	Na_v2.1
Scn8a	Na_v1.6
Scn9a	Na_v1.7
Scn10a	Na_v1.8
Scn11a	Na_v1.9
Hcn1	HCN1
Hcn2	HCN2
Hcn3	HCN3
Hcn4	HCN4
Ano1	TMEM16A
Ano2	TMEM16B
Cacna1c	Ca_v1.2
Cacna1d	Ca_v1.3
Cacna1a	Ca_v2.1
Cacna1b	Ca_v2.2
Cacna1g	Ca_v3.1
Cacna1h	Ca_v3.2
Cacna1i	Ca_v3.3
Kcnt1	SLACK
Kcnt2	SLICK
Kcnma1	BK(K_{Ca}1.1)
Kcnn1	SK1(K_{Ca}2.1)
Kcnn2	SK2(K_{Ca}2.2)
Kcnn3	SK2(K_{Ca}2.3)
Kcnn4	IK(K_{Ca}3.1)
Kcna1	K_v1.1
Kcna2	K_v1.2
Kcna3	K_v1.3
Kcna4	A-type (K_v1.4)
Kcna5	K_v1.5
Kcna6	K_v1.6
Kcna7	K_v1.7
Kcnb1	K_v2.1
Kcnb2	K_v2.2
Kcnc1	K_v3.1
Kcnc2	K_v3.2
Kcnc3	A-type (K_v3.3)
Kcnc4	A-type (K_v3.4)
Kcnd1	A-type (K_v4.1)
Kcnd2	A-type(K_v4.2)
Kcnd3	A-type(K_v4.3)
Kcnf1	K_v5.1
Kcng1	K_v6.1
Kcng2	K_v6.2
Kcng4	K_v6.3
Kcng3	K_v6.4
Kcnq2	K_v7.2
Kcnq3	K_v7.3
Kcnq4	K_v7.4
Kcnq5	K_v7.5
Kcnk1	K_{2P}1.1
Kcnk2	K_{2P}2.1
Kcnk3	K_{2P}3.1
Kcnk4	K_{2P}4.1
Kcnk5	K_{2P}5.1
Kcnk6	K_{2P}6.1
Kcnk7	K_{2P}7.1

S7

A

Immune cell Markers

Gene	Log2Fold change	Percentage 1	Percentage 2	Adjusted P value
C1qa	3.5180842	0.877	0.046	0.000000e+00
Cxcl2	3.6511131	0.743	0.027	0.000000e+00
C1qb	3.4324098	0.823	0.038	0.000000e+00
C1qc	3.3461027	0.827	0.037	0.000000e+00
Tyrobp	2.9095431	0.937	0.042	0.000000e+00
Fcer1g	2.8219276	0.930	0.037	0.000000e+00
Cfh	2.8945865	0.897	0.032	0.000000e+00
Aif1	2.6733969	0.910	0.065	0.000000e+00
Il1b	3.0137275	0.760	0.023	0.000000e+00
Bcl2a1	2.6581615	0.850	0.023	0.000000e+00
Cd83	2.7810403	0.883	0.018	0.000000e+00
Mrc1	2.4344799	0.867	0.021	0.000000e+00
Ccl6	2.3750514	0.840	0.015	0.000000e+00
Pf4	2.1622164	0.780	0.014	0.000000e+00
Laptm5	2.0547600	0.993	0.058	0.000000e+00
Cybb	2.3030469	0.893	0.014	0.000000e+00
Csf1r	2.2658411	0.880	0.016	0.000000e+00
Clec10a	2.2311328	0.870	0.018	0.000000e+00
Rgs1	1.8959000	0.790	0.012	0.000000e+00
Cfd	1.4389330	0.637	0.009	0.000000e+00

B

Vascular smooth muscle cell Markers

Gene	Log2Fold change	Percentage 1	Percentage 2	Adjusted P value
Acta2	3.4346256	0.845	0.022	0.000000e+00
Tagln	3.2154856	0.764	0.078	9.542926e-145
RGD1564664	2.9895026	0.991	0.253	1.159849e-102
Myh11	2.8686134	0.627	0.054	4.842476e-133
Rasl11a	2.6877606	0.891	0.055	8.564578e-253
Mustn1	2.5042547	0.855	0.117	1.658917e-132
Tpm2	2.4530870	0.882	0.275	2.984776e-73
Myl9	2.4404292	0.891	0.239	4.692441e-85
Mgp	2.3238439	1.000	0.211	1.751286e-112
Des	2.1621336	0.818	0.022	0.000000e+00
Vtn	2.0786987	0.855	0.060	3.201642e-213
Cox4i2	1.9366285	0.918	0.196	9.119496e-104
Rgs5	1.8996050	0.736	0.022	0.000000e+00
Tpm1	1.8941308	0.918	0.575	5.534833e-44
Fabp4	1.8823574	0.718	0.025	2.387438e-280
Adamts1	1.8798563	0.700	0.179	1.850121e-52
Mylk	1.8776962	0.836	0.091	4.514715e-153
Npy1r	1.8622683	0.855	0.031	0.000000e+00
Ndufa4l2	1.8418009	0.918	0.097	8.715348e-177
Hopx	1.7450186	0.864	0.050	5.282456e-251

C

Glia cell Markers

Gene	Log2Fold change	Percentage 1	Percentage 2	Adjusted P value
Dbi	2.0501341	1.000	0.976	0.000000e+00
Fxyd1	2.0230451	0.998	0.679	0.000000e+00
Sostdc1	1.9505253	0.968	0.292	0.000000e+00
Scn7a	1.8488933	0.990	0.387	0.000000e+00
Cdh19	1.7355892	0.980	0.265	0.000000e+00
Vwa1	1.6739017	0.992	0.472	0.000000e+00
Abca8a	1.6606951	0.994	0.604	0.000000e+00
Sfrp5	1.6566244	0.952	0.317	0.000000e+00
Art3	1.5968683	0.992	0.335	0.000000e+00
S100b	1.5535275	0.982	0.475	0.000000e+00
LOC108348061	1.5519136	0.860	0.415	0.000000e+00
Gpr37l1	1.5491717	0.945	0.199	0.000000e+00
Cnn3	1.5430965	0.995	0.760	0.000000e+00
Lgi4	1.5341173	0.979	0.311	0.000000e+00
Col28a1	1.5185676	0.912	0.200	0.000000e+00
Pdlim4	1.5179088	0.966	0.323	0.000000e+00
Tmod2	1.5000766	0.929	0.392	0.000000e+00
Gpm6b	1.4857464	0.972	0.330	0.000000e+00
Rarres2	1.4785534	0.986	0.456	0.000000e+00
Egfl8	1.4631824	0.970	0.365	0.000000e+00

D

Fibroblast Markers

Gene	Log2Fold change	Percentage 1	Percentage 2	Adjusted P Value
Apod	5.0702161	0.972	0.444	8.531948e-70
Dcn	4.4300866	1.000	0.257	1.504777e-106
Myoc	4.2646390	0.991	0.091	5.961128e-216
Lum	3.8051183	1.000	0.113	1.256168e-188
Gsn	3.0628327	1.000	0.824	3.071820e-63
Thbs4	2.9821255	1.000	0.044	0.000000e+00
Col3a1	2.9581712	1.000	0.846	9.210442e-65
Mgp	2.8940880	0.991	0.212	2.892069e-110
Igfbp5	2.6937379	0.755	0.328	2.660498e-33
Col1a1	2.6731355	1.000	0.823	4.557282e-63
Col15a1	2.5881164	1.000	0.545	6.192017e-70
Crispld2	2.5701496	0.943	0.042	0.000000e+00
Fn1	2.5640687	1.000	0.250	3.140104e-102
Gpc3	2.5392989	0.972	0.024	0.000000e+00
Smoc2	2.4793013	0.934	0.027	0.000000e+00
Serpinf1	2.4357652	0.972	0.066	6.852849e-261
Pcolce	2.4270370	1.000	0.151	6.559569e-153
Mmp2	2.3279613	1.000	0.120	2.809839e-179
Aebp1	2.2966367	0.981	0.103	3.657190e-192
Igfbp6	2.2607160	0.830	0.096	1.724846e-134

E

Endothelial cell Markers

Gene	Log2Fold change	Percentage 1	Percentage 2	Adjusted P Value
Plvap	2.6699906	0.976	0.030	0.000000e+00
Aqp1	2.5668906	0.961	0.146	0.000000e+00
Rgs16	2.5153660	0.821	0.101	0.000000e+00
Sele	2.4944279	0.374	0.014	7.421128e-235
Flt1	2.3509513	0.966	0.019	0.000000e+00
Slco1a4	2.1993135	0.918	0.029	0.000000e+00
Selp	2.1762584	0.466	0.018	4.578829e-295
Emcn	2.1625471	0.978	0.038	0.000000e+00
Abcg2	2.1382139	0.913	0.041	0.000000e+00
Cldn5	2.1186817	0.787	0.024	0.000000e+00
Cyyr1	2.1040015	0.971	0.028	0.000000e+00
Fam110d	2.0985356	0.886	0.022	0.000000e+00
Rnd1	2.0177000	0.659	0.071	2.776598e-287
Cav1	2.0059116	0.957	0.087	0.000000e+00
Cxcl12	1.9573106	0.717	0.027	0.000000e+00
Thbd	1.9078395	0.882	0.118	0.000000e+00
Cdh5	1.8601120	0.966	0.029	0.000000e+00
Adgrf5	1.8066178	0.940	0.050	0.000000e+00
Id1	1.7850078	0.812	0.168	4.762163e-249
Kdr	1.7653330	0.860	0.018	0.000000e+00

F

Sympathetic neuron Markers

Gene	Log2Fold change	Percentage 1	Percentage 2	Adjusted P Value
Snhg11	3.2061663	0.990	0.046	0.000000e+00
Insrr	1.7951871	0.997	0.062	0.000000e+00
Smpd3	0.8982419	0.895	0.085	0.000000e+00
Cacna1b	1.1997503	0.978	0.075	0.000000e+00
Spock3	0.7479784	0.762	0.078	8.747048e-264
Gpr22	0.5700290	0.790	0.054	0.000000e+00
Tmem59l	0.7677566	0.787	0.072	0.000000e+00
March11	0.6499392	0.857	0.064	0.000000e+00
Sgsm1	0.9615743	0.956	0.057	0.000000e+00
Atp2b2	0.8224142	0.908	0.061	0.000000e+00
Ptchd1	0.8112243	0.838	0.063	0.000000e+00
Dmkn	0.6906363	0.698	0.054	4.118108e-285
Eml5	1.0585886	0.911	0.036	0.000000e+00
Gria2	1.2867993	0.962	0.054	0.000000e+00
Arfgef3	0.6022328	0.883	0.036	0.000000e+00
Slc27a6	0.4985297	0.581	0.034	2.999406e-273
Slc7a14	0.6440851	0.825	0.064	0.000000e+00
B3galt1	0.6604370	0.854	0.050	0.000000e+00
Plppr5	0.5191926	0.794	0.037	0.000000e+00
Shisal1	0.4718486	0.816	0.044	0.000000e+00
